# Supplementary material for: Canalisation and plasticity on the developmental manifold of Caenorhabditis elegans
Source: Mol Syst Biol. 2023 Oct 18;19(11):e11835. doi: 10.15252/msb.202311835 (PMC10632735; doi:10.15252/msb.202311835)
Supplement: Supplementary file 1 — Appendix [file MSB-19-e11835-s007.pdf]

APPENDIX

Canalisation and plasticity on the developmental manifold of *Caenorhabditis elegans*

Table of Contents

Projection operators	Page 1
Projection in the context of developmental trajectories	Page 2
Appendix Figure S1	Page 2
Appendix Figure S2	Page 3
Parallel and orthogonal projections in relation to plasticity and canalisation	Page 3
Appendix Figure S3	Page 4
Notes on other dimensionality reduction methods	Page 4
Appendix Figure S4	Page 5
Fraction on-food analysis	Page 5
Appendix Figure S5	Page 6
Appendix References	Page 6

Projection operators

In the context of this work, projection refers to a general class of idempotent functions (maps) from a set (space) into a subset (subspace). Idempotent means that repeated applications after the first do not further alter the result. If P is a projection map, this means that $P\big(P(x)\big) = P(x)$ In particular we are interested in projections in Euclidean spaces (linear projections and projections onto manifolds) and also projections in Hilbert spaces. The most familiar projection operator may be the projection onto the canonical basis in the n-dimensional Euclidean space $\mathbb{R}^n$, which is a linear operator. If we have a vector $\vec{v}$ in $\mathbb{R}^n$, the projection map is the inner (dot) product with the canonical basis. For example, the projection onto the "x" axis is given by:

$$P_{e_1}(\vec{v}) = \vec{v} \cdot e_1 = \vec{v} \cdot [1,0,0,0\ldots] = v_1$$

In a Hilbert space of functions, we have an analogous projection function. This is easiest to demonstrate if we have an orthonormal set of basis functions, for example, the periodic basis of the Fourier transform. In this case, the projection is still given by the inner product, but the inner product has a different form,

$$f \cdot g = \langle f, g\rangle = \frac{1}{2\pi}\int_0^{2\pi} f(x)g^*(x)dx$$

We can think of any function as a vector in this space. If we call the set of basis functions, ϕ_i then any function p can be represented as a vector of weights $p = \sum_{i=1}^{\infty}(w_i\phi_i)$ with the weights given by $w_i = \langle p, \phi_i\rangle$. We can then further 'project' this function onto a subset of basis functions using the standard dot product with the weight vector w_i. In practise, because we are using an orthonormal basis, this is equivalent to truncating the infinite sum. This projection is often required in real-world problems as the weight vector is in principle infinite dimensional and must be approximated by a finite number of basis functions. Projection operators on infinite-dimensional Hilbert spaces may seem removed from biological problems, but they arise in statistical mechanics in physical problems that are similar in some respects to biological problems. An example is the diffusion of a drop dye in water. In principle, the dynamics that govern the time evolution of the dye concentration as a function of depends on all the position and momenta of the 10^{23}particles involved. However, the dynamics of the concentration can be

described well as a function of the concentration itself. This derivation can be made exactly using projection operators (See e.g. Zwanzig R *Nonequilibrum Statistical Mechanics* Oxford University Press, 2001).

Projection in the context of developmental trajectories

For the NLPCA analysis, a 5-vector consisting of the logistic fit parameters and developmental durations $(l_{max}, r, A, t_{dev}, t_{hatch})$. This was compressed into a 3-vector (ϕ_1, ϕ_2, ϕ_3) using the NLPCA procedure.
In order to demonstrate how you can feed logistic parameters in to perform a PCA, let me provide a concrete example using a 2d ambient space, a 1d manifold, and linear PCA. Let us start with a collection of growth curves generated by the logistic function with parameters (l_{max}, r, A). For simplicity, I will fix the A parameter in all of the growth curves. Because these two parameters are correlated, we can perform PCA and reduce the 2-vector (l_{max}, r) to a single scalar ϕ_1 by projecting it onto the first principal component.

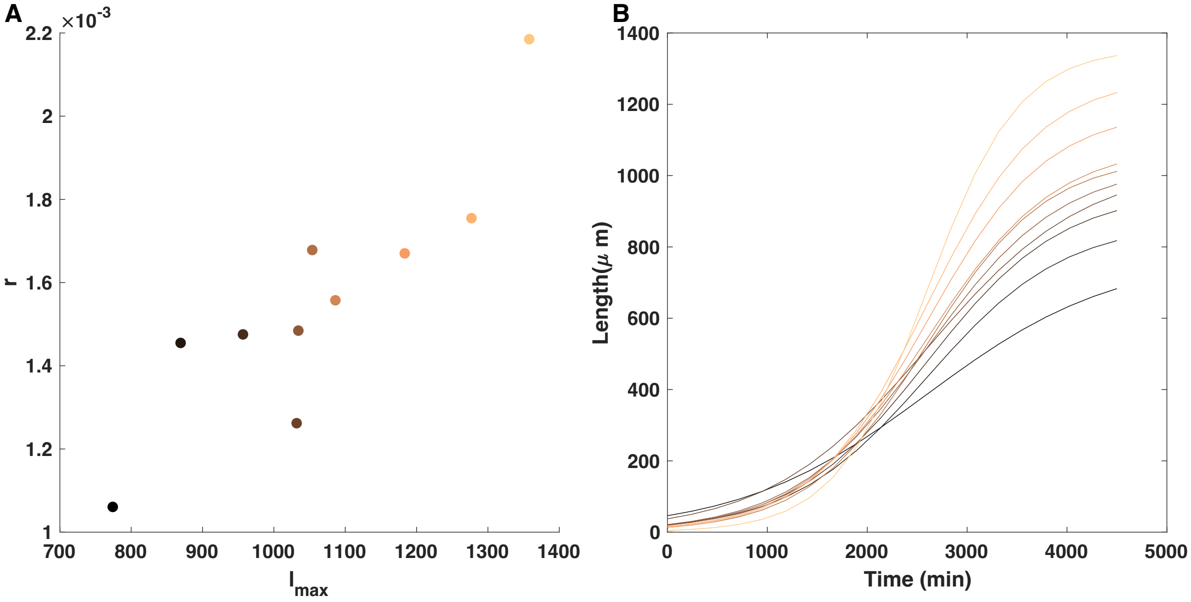

Appendix Figure S1

A. Hypothetical data for values of l_{max} and r are shown, these values are not IID random variables, but rather are chosen to be positively correlated.
B. The resulting logistic curves that are given by the l_{max} and r values with a fixed A (2600) for all curves

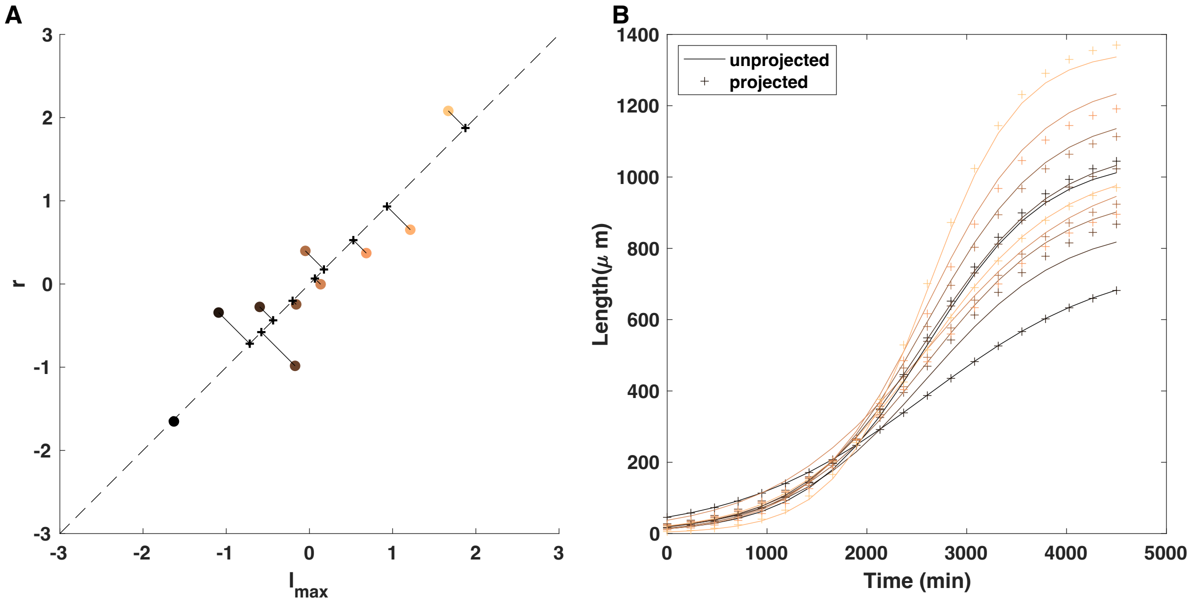

Appendix Figure S2

A. The same data as in App Fig S1A but centred and scaled for PCA. The first principal component gives the 1d manifold onto which the variation is projected. Projections are indicated by black lines and pluses.
B. The reconstructed growth curves from the projected points are shown as pluses. They correspond to the growth curves from the unprojected data of the same colour (solid lines).

The main eigenvector (first principal component) is shown by the dashed line and points in the [1,1] direction, $v = \left(\sqrt{(2)}, \sqrt{(2)}\right)$. The z-scores of data points (centred scaled data) are shown in the same colours as above. Their projections onto the first principal component are shown by the pluses.

l_{max}	r	ϕ_1	Reconstructed l_{max}	Reconstructed r
774.1	1.061e-03	-1.6404	772.1	1.064e-03
869.2	1.455e-03	-0.7174	935.4	1.342e-03
956.6	1.475e-03	-0.4364	985.2	1.427e-03
1031.9	1.262e-03	-0.5782	960.1	1.384e-03
1034.3	1.484e-03	-0.2021	1026.7	1.497e-03
1053.8	1.678e-03	0.1746	1093.3	1.611e-03
1086.2	1.557e-03	0.0657	1074.0	1.578e-03
1183.4	1.670e-03	0.5274	1155.8	1.717e-03
1276.9	1.755e-03	0.9322	1227.4	1.839e-03
1357.8	2.185e-03	1.8748	1394.2	2.123e-03

The results of reconstructing the growth curves from the compressed representation are shown by the "-+' lines in the right panel.

Parallel and orthogonal projections in relation to plasticity and canalisation

Imagine that we have a molecular process that generates values for Cx and Cy and which converts those signals into a developmental trajectory. We have generated some 'expression levels' of Cx and Cy, and these are shown in the scatter plot in App Fig 1A as filled circles. In this example, the projection operator is the linear projection on the vector pointing in the (1,1) direction, and the projection expression is shown as a black plus. Suppose a mutation changes a *wilt-type* expression pattern (App Fig 1A, green triangle) into an aberrant expression pattern (App Fig 1A, red square). In the absence of projection, or of concentration of dimension as we have called it, this aberrant expression pattern would result in a very different developmental trajectory (App. Fig 1B, red line with squares). However, under the action of the projection function, this aberrant expression point is mapped to its location on

the manifold (in this case the line Cy=Cx) which gives the reduced dimensional representation (App Fig 1A, blue diamond). The action of this projection operator results in a growth curve (App Fig 1B, blue line with diamonds) this is much less aberrant. Thus, projection onto the manifold has endowed the system with robustness. However, the projection has not completely buffered the development trajectory of this mutation. If we look at the difference between the wild-type developmental trajectory (App Fig 1B, green line with triangles), and the projected mutant developmental trajectory (App Fig 1B, blue line with diamonds), there is still variation that corresponds the component of the projection along the manifold, as shown by the purple arrow. Thus, projection parallel to the manifold allows for plasticity, but the nature of this plasticity is constrained. The removal of the component orthogonal to the manifold constrains it, and this results in robustness. The total variation that would have been produced is decomposed into the variation *along* the manifold, which is retained and gives plasticity, and the variation *orthogonal* to the manifold, which is buffered and gives canalisation.

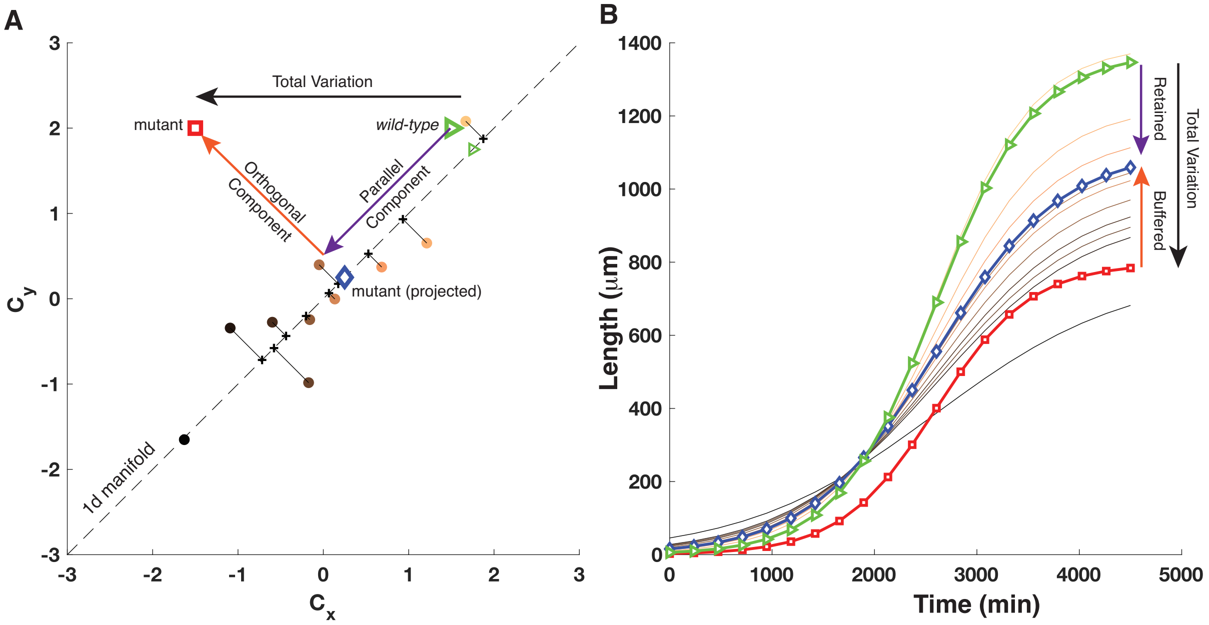

Appendix Figure S3

A. We reimagine the axes of App Fig2A as expression levels for genes controlling the parameters, Cx and Cy. Here, we show a *wild-type* expression pattern (green triangle) and its projection onto the manifold (small green triangle). A mutation may change the expression of Cx greatly but not Cy (red square), the black arrow represents this expression change. However, this new expression pattern will be projected onto the manifold (blue diamond). Thus, the total variation is decomposed into a parallel component (purple arrow) and an orthogonal component (orange arrow).
B. When these expression levels are converted back into growth curves, we see that the mutation should have resulted in a very aberrant growth cure (red line with squares). However, because of the projection, the actual growth curve for this expression pattern is less aberrant (blue line with diamonds). The difference between these is the buffered variation (orange arrow). However, the variation induced by the mutation is not completely lost, the portion that is projected (purple arrow) results in the difference from the *wild-type.*

Notes on other dimensionality reduction methods

We chose NLPCA because we did not expect there to be linear relationships between the phenotypic parameters we measured. When we first looked at the 3d scatter plot of logistic fit parameters, it looked like a curved plane. Some of the simplest physical laws in thermodynamics, for example the ideal gas law, have a nonlinear relationship between the variables of interest, i.e. $PV = nRT$ for P, V and T. With this in mind and because NLPCA is more general and is able to find linear relationships as well if they exist, we chose this over PCA. It does seem that there

is some non-linear relationship between the variables, as NLPCA performs better than regular PCA, as shown in the cumulative variance explained plot below.

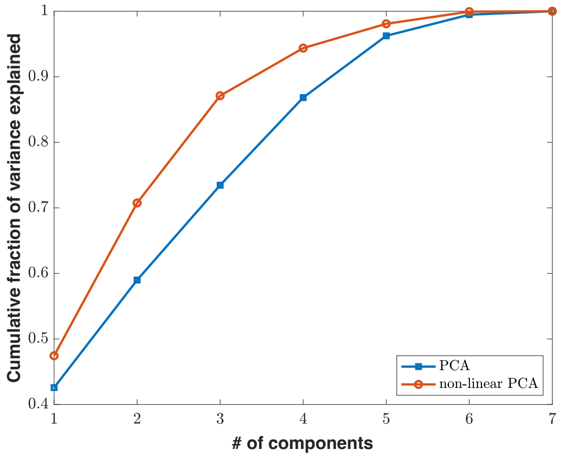

Appendix Figure S4

A. Cumulative fraction of variance explained using with linear PCA (blue) or nonlinear PCA (orange) to reduce the dimension of an extended dataset of 7 growth and development parameters. As the number of principal components increases, cumulative variance explained goes up, however, nonlinear PCA performs better as indicated by the orange curve being above the blue curve.

We did not choose t-SNE because it is not density preserving. This is clearly presented by Wattenberg *et al.* (Wattenberg, et al., "How to Use t-SNE Effectively", Distill, 2016. http://doi.org/10.23915/distill.00002) which I quote below.

> The t-SNE algorithm adapts its notion of "distance" to regional density variations in the data set. As a result, it naturally expands dense clusters, and contracts sparse ones, evening out cluster sizes. To be clear, this is a different effect than the run-of-the-mill fact that any dimensionality reduction technique will distort distances. Rather, density equalization happens by design and is a predictable feature of t-SNE.

Because we were particularly interested in how the data clustered after dimensionality reduction, we decided not to use t-SNE.

The UMAP technique relies on an assumption we did not believe to be true for our data, i.e., that the data are uniformly distributed on Riemannian manifold, although it is difficult to assess how close to uniformly distributed the data truly are. In addition, while I can draw mathematical analogies between projection operators and both PCA and NLPCA, the relationship between projections and t-SNE or UMAP were not clear to me.

Fraction on-food analysis

The development of *C. elegans has been noted to be more rapid* in environments where they are completely surrounded by food than on plates where they can be in or out of food. It is also known that the tendency for animals to be on food varies among strains (Chang *et al*, 2006; Bretscher *et al*, 2008). In these experiments, animal behaviour can influence growth, as the experimental design provides an opportunity for the worms to leave and enter the food patch. Microfluidic chamber-based systems are another alternative (Uppaluri & Brangwynne, 2015) in which bacterial density can be precisely controlled; however, the aqueous environment can result in 'thrashing' behaviour, which has been shown to affect animal physiology (Laranjeiro *et al,* 2019). Because in our systems, animals can freely move on and off food, it is possible that behavioural differences could explain developmental changes. To assess this, we quantified whether animals that spend more time in food were more likely to develop more quickly. We find that this does not seem to be the case as there is no significant correlation between developmental duration and the fraction of time the animals were found in food (App Fig S5A). In addition to individuals, the average development time was calculated for each combination of environment and genotype and

plotted against the total fraction of time spent by all individuals on food, and this also does not show a significant correlation (App Fig S5B).

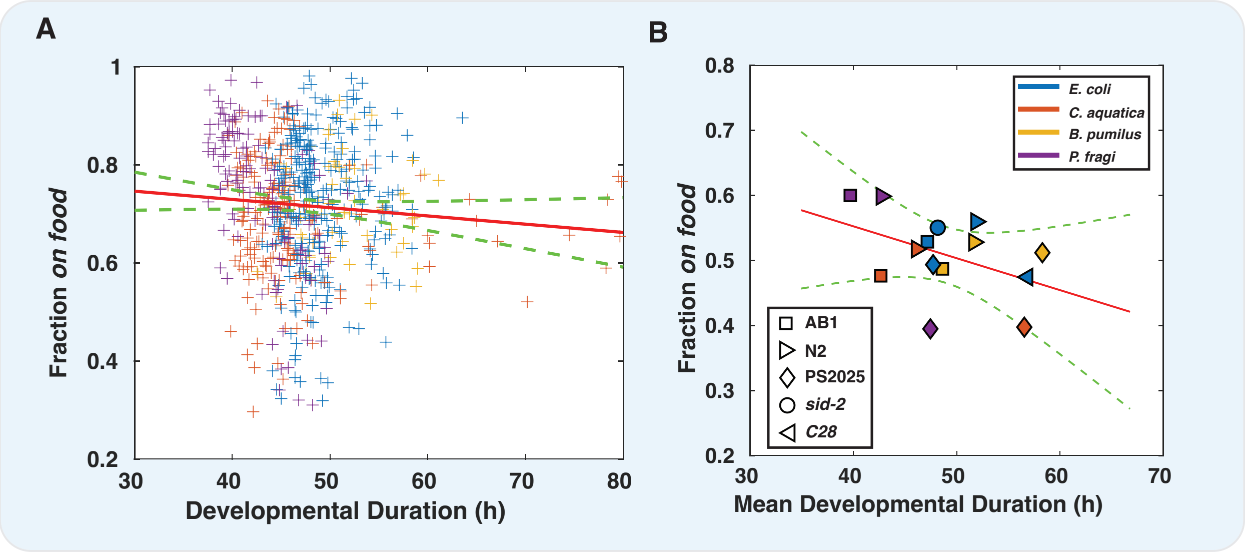

Appendix Figure S5

A. For each individual, the fraction on-food is plotted against that individual's developmental duration. Fraction on-food is the fraction of frames the animal id recorded less than 1.33mm from the center of the mini well. The best fit linear regression (red line) with is shown with its 95% confidence interval (green dashed lines). Colour indicates the individual's diet, as indicated in the legend in B.

B. Mean fraction on-food and the mean developmental duration for all individuals in a condition. The best fit linear regression (red line) with is shown with its 95% confidence interval (green dashed lines). The slope of the regression is not statistically significantly different from 0 for either the individuals or the means in each condition. Colours represent the type of bacterial food for those individuals and symbols represent the animals genotype.

Appendix References

1. Chang AJ, Chronis N, Karow DS, Marletta MA & Bargmann CI (2006) A distributed chemosensory circuit for oxygen preference in C. elegans. *PLoS Biol* 4: e274
2. Bretscher AJ, Busch KE & de Bono M (2008) A carbon dioxide avoidance behavior is integrated with responses to ambient oxygen and food in *Caenorhabditis elegans*. *Proc Natl Acad Sci U S A* 105: 8044–8049
3. Uppaluri S & Brangwynne CP (2015) A size threshold governs Caenorhabditis elegans developmental progression. *Proc Biol Sci* 282: 20151283
4. Laranjeiro R, Harinath G, Hewitt JE, Hartman JH, Royal MA, Meyer JN, Vanapalli SA & Driscoll M (2019) Swim exercise in *Caenorhabditis elegans* extends neuromuscular and gut healthspan, enhances learning ability, and protects against neurodegeneration. *Proc Natl Acad Sci U S A* 116: 23829–23839
