# Supplementary material for: Canalisation and plasticity on the developmental manifold of Caenorhabditis elegans
Source: Mol Syst Biol. 2023 Oct 18;19(11):e11835. doi: 10.15252/msb.202311835 (PMC10632735; doi:10.15252/msb.202311835)
Supplement: Supplementary file 2 — Expanded View Figures PDF [file MSB-19-e11835-s003.pdf]

Expanded View Figures

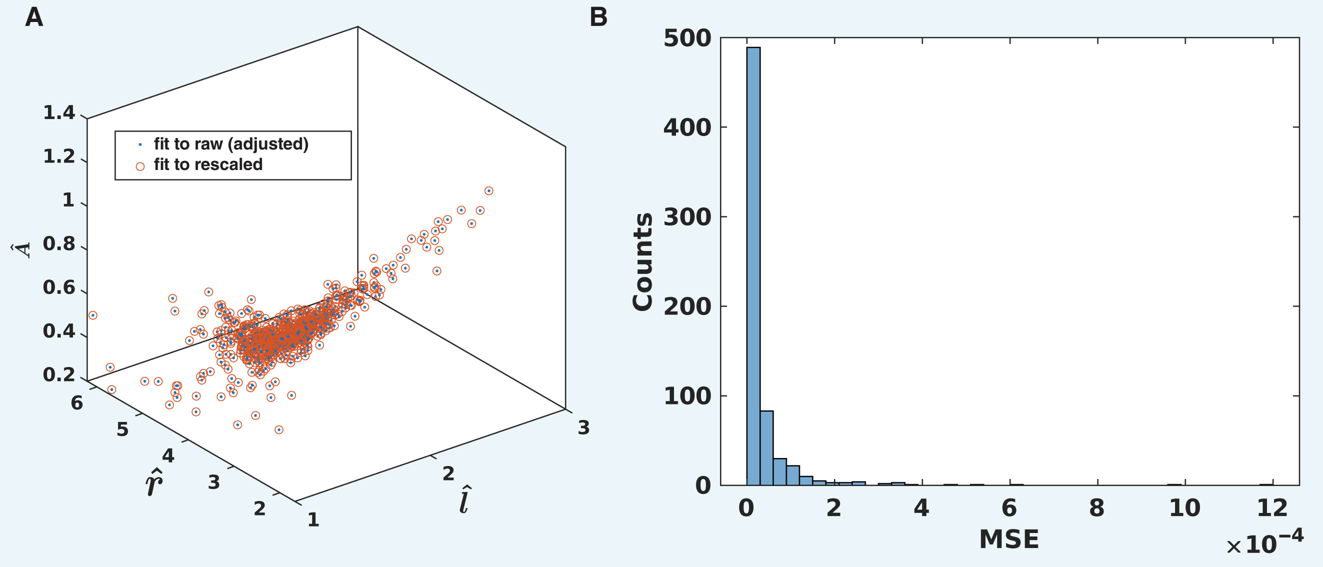

Figure EV1. Rescaled logistic fit parameters are equivalent to fits performed on rescaled data.

A Scatter plot of the three logistic fit parameters for each curve that were obtained either by fitting the raw data directly and renormalising the fit parameters with respect to the development time (blue points) or rescaling the data and then performing the fit (orange points).

B Histogram of the mean squared error between the raw adjusted and the rescaled fit data points.

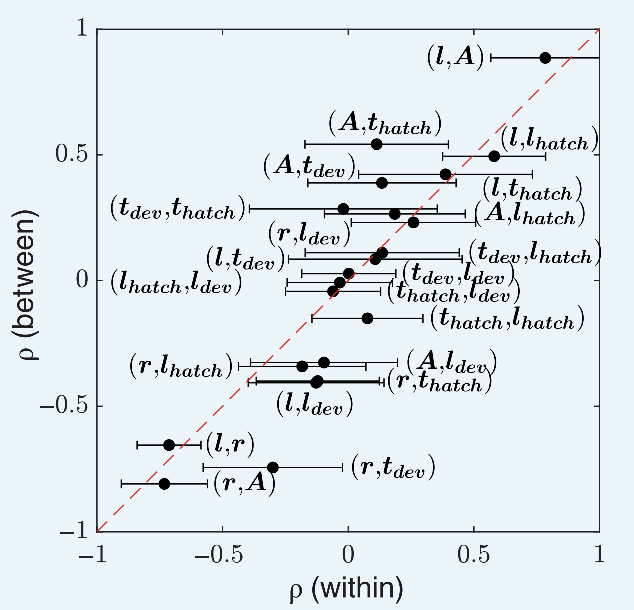

Figure EV2. Detailed within-population vs. between-population trait pair correlations.

Trait pairs within- vs. between-population correlations are plotted for an extended collection of traits (21 pairs of 7 traits) c.f. Fig 3E (10 pairs of 5 traits). The mean of the within-population correlation coefficients is plotted with the standard deviation as error bars against the between-population correlation coefficient. The red dashed line indicates equivalence. N = 673 biological replicates for each trait pair. The pairs of traits are annotated on the figure. In addition to (l, r, A, t_{dev}, t_{hatch}) shown in Fig 3E, this plot also includes l_{hatch} the length of the newly hatched animal, and l_{dev}, the length of the adult animal when it lays its first egg.

© 2023 The Authors
