# Supplementary material for: Canalisation and plasticity on the developmental manifold of Caenorhabditis elegans
Source: Mol Syst Biol. 2023 Oct 18;19(11):e11835. doi: 10.15252/msb.202311835 (PMC10632735; doi:10.15252/msb.202311835)
Supplement: Supplementary file 3 — PDF+ [file MSB-19-e11835-s008.pdf]

Article

SOURCE DATA TRANSPARENT PROCESS OPEN ACCESS

molecular systems biology

# Canalisation and plasticity on the developmental manifold of *Caenorhabditis elegans*

David J Jordan* & Eric A Miska

## Abstract

**How do the same mechanisms that faithfully regenerate complex developmental programmes in spite of environmental and genetic perturbations also allow responsiveness to environmental signals, adaptation and genetic evolution? Using the nematode *Caenorhabditis elegans* as a model, we explore the phenotypic space of growth and development in various genetic and environmental contexts. Our data are growth curves and developmental parameters obtained by automated microscopy. Using these, we show that among the traits that make up the developmental space, correlations within a particular context are predictive of correlations among different contexts. Furthermore, we find that the developmental variability of this animal can be captured on a relatively low-dimensional *phenotypic manifold* and that on this manifold, genetic and environmental contributions to plasticity can be deconvolved independently. Our perspective offers a new way of understanding the relationship between robustness and flexibility in complex systems, suggesting that projection and concentration of dimension can naturally align these forces as complementary rather than competing.**

**Keywords** canalisation; dimensionality reduction; phenotypic manifold; plasticity
**Subject Category** Development
**DOI** 10.15252/msb.202311835 | Received 21 June 2023 | Revised 26 September 2023 | Accepted 5 October 2023 | Published online xx xx 2023
**Mol Syst Biol. (2023) 19: e11835**

## Introduction

Biological systems are remarkable for their ability to generate reproducible macroscopic dynamics from the complex interactions of large numbers of microscopic components. For example, in animals, the development of an entire organism from a single cell proceeds faithfully each generation even in the presence of environmental fluctuations and molecular noise. Such robustness arises at many spatial and temporal scales, for example, gene expression patterns give rise to reproducible cell differentiation (Rulands & Simons, 2017) neural and muscular activity generate locomotion (Stephens *et al*, 2008) and interactions between individuals of different species give rise to surprisingly reproducible ecological dynamics (Frentz *et al*, 2015). This robustness is called *canalisation* (Waddington, 1942), and dynamics that are canalised are said to be *homeorhetic* (Waddington, 1957; Chuang *et al*, 2019), terms introduced by CH Waddington in his series of foundational works on the development.

Although robust, canalised processes nevertheless allow for important variability; stem cell populations generate diverse tissue types, behaviours respond to stimuli and environmental cues, and populations adapt to changing environments. However, the structure of this macroscopic variability is often much more constrained than the intrinsic variability of the microscopic processes that underlie it. Although gene expression determines the dynamics of the cell cycle, variations in the cell cycle timing are largely insensitive to stochastic fluctuations in the levels of thousands of proteins. Thus, the ways in which these macroscopic phenotypes can vary are relatively "low-dimensional" compared with the ways in which the individual components can vary.

A low-dimensional manifold is a locally Euclidean subspace of a higher-dimensional space. We call this higher-dimensional space the "ambient" space. For example, the surface of a sphere is a two-dimensional manifold embedded in a three-dimensional ambient space. In the ambient space, the points on the sphere could be identified by their three coordinates ($x$, $y$, $z$; with the added condition that $\sqrt{(x^2+y^2+z^2)}=r$). However, we can also describe coordinates on the surface of the sphere using a 2d coordinate system, such as their latitude $\phi$ and longitude $\gamma$. These coordinates are given by the nonlinear projection map $\phi = sin^{-1}\left(\frac{z}{r}\right)$ and $\lambda = \mathrm{atan2}\left(\frac{y}{x}\right)$. In this procedure, variations in elevation will be lost, as the projection map admits only a fixed $r$; in this sense, the projection reduces variation. An example of a projection in a biological system is the gene regulatory dynamics that map the high-dimensional ambient space of gene expression onto the subspace of cell identities. In this work, we introduce the term *concentration of dimension* to differentiate this phenomenon from numerical and computation methods which extract low-dimensional representations, collectively referred to as dimensionality reduction techniques.

We propose that projection of variations into a lower-dimensional space may provide a way for biological systems to be both robust and flexible. Robustness arises because most variations manifest as excitations onto relatively few phenotypic modes, whereas flexibility is permitted along these modes. As an example,

Department of Biochemistry, University of Cambridge, Cambridge, UK
*Corresponding author. Tel: +44 7484 872451; E-mail: dj333@cam.ac.uk

© 2023 The Authors. Published under the terms of the CC BY 4.0 license

consider how facial diversity can be generated by the combination of relatively few *eigenfaces* (Turk & Pentland, 1991). Here, the eigenfaces are the modes in this system, and varying the weights of each mode can capture many diverse faces. However, even large variations in these weights are unlikely to produce nonface images. Low-dimensional representations of phenotypic diversity are ubiquitous in living systems. Variations in phenotypes that tend to a stable state during development have been successfully represented in low-dimensional phenotypic spaces called "morpho-spaces," for example, morphological traits such as the arrangement of flowers in a plant (Prusinkiewicz *et al*, 2007), the shapes of finch beaks (Campàs *et al*, 2010), of coiled sea shells (Raup, 1966), and even the shape of the influenza antigen (Smith *et al*, 2004). Furthermore, while the dimensionality of dynamic, time-varying and responsive phenotypes is more difficult to define rigorously (Bialek, 2022), it has been shown in some cases to be low-dimensional. Some recent examples include the crawling behaviour of *C. elegans* (Costa *et al*, 2023) and the neuronal dynamics that underlie it (Brennan & Proekt, 2019), the swimming behaviour of both eukaryotic (*Tetrahymena thermophila*; Jordan *et al*, 2013) and prokaryotic (*Escherichia coli*) single-celled organisms (Pleška *et al*, 2021), and the transcriptional trajectories of cells during fate determination (Huang *et al*, 2005). Concentration of dimension may be an intrinsic property of systems where robustness and control of the macroscopic observables are required (Eckmann & Tlusty, 2021) and may arise from constraints imposed by steady-state growth (Klumpp & Hwa, 2014; Kaneko & Furusawa, 2021), or trade-offs between phenotypic archetypes suited for different tasks (Shoval *et al*, 2012).

Here, we propose that the process of canalisation may be superseded by a more general process of phenotypic concentration of dimension. That is, genetic networks evolve such that most variations will be projected onto a low-dimensional manifold, and this in turn provides both canalisation and phenotypic plasticity. The concentration of dimensions manifests itself as the covariation of traits in a high-dimensional multiphenotype space. This structure of covariation is called *phenotypic integration* (Pigliucci, 2003), and its geometric properties can be captured by a phenotypic manifold, that is, a lower-dimensional space that faithfully captures most of the variation observed in a higher-dimensional space. The process of finding such a manifold is called dimensionality reduction. The determination of this space and how it is measured can be essential to finding the dominant modes of phenotypic variation and thus to assessing the degree of canalisation. A recent example comes from the developmental programme of the wing of the fruit fly *Drosophila melanogaster* (Alba *et al*, 2021), which used landmark free morphometrics to uncover a dominant mode of variation that was not apparent from traditional landmark-based techniques.

In this work, we use a custom-built automated imaging system to map the phenotypic space of growth and development of *C. elegans*. Using this system, we recorded 673 individual growth curves during the ~70 h of their development from eggs to reproductive adults and manually recorded the timing of egg hatching and reproductive maturation in single animals. To construct as complete a manifold as possible, we sampled extant variations in different genetic and environmental contexts, including both natural genetic variation and single-gene mutants. Although the most unbiased approach to sampling genetic variation would be random mutagenesis, due to the immense sampling space, efficient sampling cannot be done. For this reason, we chose to sample "wild isolates" of *C. elegans*, that is, strains collected from nature and whose collections of genetic changes have been subjected to natural selection. For environmental diversity, we chose to alter the animal's diet; *C. elegans* feed on bacteria, and we chose a collection of bacteria that included the standard laboratory bacteria *E. coli* as well as bacteria isolated from natural sites where *C. elegans* were also collected (Frézal & Félix, 2015; Samuel *et al*, 2016). Measurements were taken for three wild isolates of *C. elegans*, each fed one of four different bacterial diets, and two mutants of the laboratory strain N2.

Using these data, we demonstrate that the space of developmental trajectories can be captured by a low-dimensional manifold and show a correspondence between the directions of fluctuations in a fixed context and the directions in which populations will shift due to genetic or environmental changes. We find that the manifold obtained using nonlinear dimensionality reduction techniques captures developmental variations in a way that allows one to neatly decompose the contributions of genetics and environment independently, with the major mode of variation corresponding to environmental shifts and the second mode corresponding to genetic changes.

## Results

Characteristics that change over time are more difficult to quantify and compare than those that are static or near a steady state. Often, dynamic phenotypes such as these are measured and compared at a single fixed time, but in this case, proper alignment or synchronisation can be challenging. Ideally, one would like to compare the time series of time-varying phenotypes and compare these directly. The growth of a multicellular organism is a good example of such a time-dependent phenotype. Ideally, one would like to compare growth curves aligned to an unambiguous starting time.

To this end, we designed and constructed a low-cost parallel imaging platform capable of measuring *C. elegans* growth for 60 individual animals simultaneously over the course of their ~70 h development at a temporal resolution of ~0.001 Hz, resulting in a time series of ~200 observations per animal. In addition to length and area measured automatically, egg hatching and first egg laying by mature adults are manually recorded. These not only provide estimates of the animal's reproductive development but also provide a fixed time to which the time series can be aligned. Reproductive development is generally correlated with growth, but it does not necessarily have to be. Because animals grow from approximately 0.2 to 1 mm in length during their lifetime, any wide-field system capable of imaging many isolated worms simultaneously would lack the necessary resolution. To solve this, we developed a system that uses a fixed lens and USB camera that is programmed to move between 6-mm-diameter custom-made wells using an XY-plotting robot. Sample images from the time series are shown (Fig 1A; inset, 1–8), along with the associated time series and the best-fit logistic function of the form:

$$l(t) = \frac{l_{max}}{1 + e^{-r(t-A)}} \quad (1)$$

as determined by nonlinear least squares fitting, giving three parameters for each curve (Fig 1E). We used this system to record

 © 2023 The Authors

development in a collection of *C. elegans* isolated from the wild and fed on various bacteria, some of which were collected from sites where *C. elegans* were found. In total, we assayed five unique *C. elegans* genotypes, three wild isolates (Fig 1B) and two N2 mutants, each fed on four different bacterial diets (Fig 1C) and collected a total of 673 growth curves (Fig 1D), in addition to developmental data, across these conditions. The developmental data consist of the duration of *ex utero* embryonic development and the duration of reproductive maturation. These are measured as the time from egg-laying to egg-hatching $t_{hatch}$ and the time from hatching until the animal grows to reproductive age and lays its first egg $t_{dev} = (t_{laid} - t_{hatch})$ as shown in Fig 1A. While these durations were single scalar quantities, the growth curves consisted of hundreds of points. Dimensionality reduction could have been performed directly on these high-dimensional vectors after appropriate regularisation, but instead these growth curves were fit with the logistic function (equation 1) which performed similarly well and whose parameters are easier to interpret (see Fig EV1). The three parameters, which we call the maximum length $l_{max}$, the growth rate $r$ and the shift $A$, determine the saturation value of the growth curve, the growth rate at the inflection point and the temporal shift of the inflection point, respectively.

For each growth curve, we have an independent measurement of the reproductive development time $t_{dev}$ and of the animal's length at this time $l(t_{laid}) = l_{dev}$ (Fig 2A, squares). We can use these additional parameters to rescale each growth curve. First, we divide the length as a function of time by $l_{dev}$, yielding:

$$\hat{l}(t) = \frac{l_{max}/l_{dev}}{1 + e^{-r(t-A)}}$$

Then, rescaling time $t \to t_{dev}$

$$\hat{l}(\hat{t}) = \frac{l_{max}/l_{dev}}{1 + e^{-rt_{dev}\left(\hat{t}-A/t_{dev}\right)}} \tag{2}$$

Thus, the normalisation simply rescales the fit parameters from $[l_{max}, r, A] \to [l_{max}/l_{dev}, r{\cdot}t_{dev}, A/t_{dev}]$ yielding unit-less quantities for the fit parameters. The fitting procedure on the normalised curves recovers the rescaled fitting parameters from the unnormalised data (see Fig EV2). Interestingly, we find that rescaling the curves to an independently measured parameter, the reproductive developmental duration, seems to collapse the growth curves, consistent with temporal scaling observed previously (Filina *et al*, 2022), for example (Fig 2B). To compare the variance of all the rescaled growth curves with the variance of the nonrescaled growth curves, the raw data were normalised to the mean duration of development and the mean and standard deviation of the resulting growth curves were compared, showing that the rescaled growth curves "collapse" and that their resulting standard deviation is smaller than for normalised raw data (Fig 2C).

The growth curves of *C. elegans* of different genetic backgrounds and grown on different food sources differ in their maximum length, their growth rate and in the shift, as well in the durations of *ex-utero* development and reproductive development. However, we find that these parameters do not vary independently. For example, some recent work has shown a negative correlation between growth rate and developmental duration in *C. elegans* and we also observe this in our data. They suggest that this may be a mechanism to reduce the variability in adult size in Stojanovski *et al* (2022). If correlations between traits arise from a mechanism for control such as this, we would expect variation within populations to be correlated in the same way as variations between populations. Strikingly, we find that if parameters are correlated among individuals in one context, these correlations are more likely to also appear between populations in different contexts (To see how such correlations may arise, refer to Box 1). For example, there is a strong negative correlation between the maximum length and the growth rate among individuals in all combinations of conditions. Likewise, the mean values of these parameters among populations grown in different conditions are also negatively correlated (Fig 3A and B). In contrast, the length and the duration of reproductive development are not strongly correlated among individuals, and neither are they correlated between populations (Fig 3C and D). Computing the correlation coefficient (ρ) among individuals in a sample in fixed conditions and plotting it against the value computed between-population means among different conditions shows that this seems to be true in general for different pairs of parameters of *C. elegans* development (Figs 3E and EV2).

The geometric structure of these correlations can be captured by a low-dimensional manifold in the ambient phenotypic space (Appendix Figs S1 and S2). Nonlinear principal component analysis (Scholz *et al*, 2005) was used to determine the shape of this manifold from the ambient space of rescaled logistic parameters with the developmental durations included (Fig 4A). The first two nonlinear principal components capture 93% of the variance. While this dimensionality reduction may seem modest, in fact, the true dimensionality of the growth curve space is higher, some of the dimensions having already been compressed by the logistic fit. Within this embedding of growth curves, animals tend to cluster in $\phi_1$ according to their food source (Fig 4B) and in $\phi_2$ according to their genetic background (Fig 4C; note only natural genetic variants are shown). This decomposition can be quantified with a linear regression model predicting either the nonlinear principal component embedding $(\phi_1, \phi_2)$ or the rescaled logistic fit parameters $\left(\hat{l}, \hat{r}, \hat{A}\right)$ using the genetic or environmental conditions as independent variables. Linear regression models of the form:

$$y(G, E) = \beta_0 + \textstyle\sum_i \beta_{1,i} G + \sum_j \beta_{2,j} E + \in$$

were fit where $\in$, with $y \in \left(\phi_1, \phi_2, \hat{l}, \hat{r}, \hat{A}\right)$, $G$ and $E$ are indicator variables which take the value of 1 or 0 depending of the strain $i$ and food $j$ in that condition, and $\in$ are the residuals to be minimised. In Fig 4B and C, the distributions grouped by environment and genotype, respectively, have different means. We can quantify this using the F-statistic, which is the variance of the between-group means divided by the mean of the within-group variances. Using this, we can see that grouping the data by environment does in fact give distinct distributions in $\phi_1$ and grouping by genotype gives distinct distributions in $\phi_2$ (Fig 4E). However, a linear regression of the parameters before dimensionality reduction does not give a clean separation between genotype and environment.

The eigenfunctions of the nonlinear principal component analysis cannot be derived analytically but can be investigated by varying each component while keeping the others fixed. If $\phi_2$ is fixed $(\phi_2 = 0)$, the shapes of the resulting growth curves as $\phi_1$ is varied

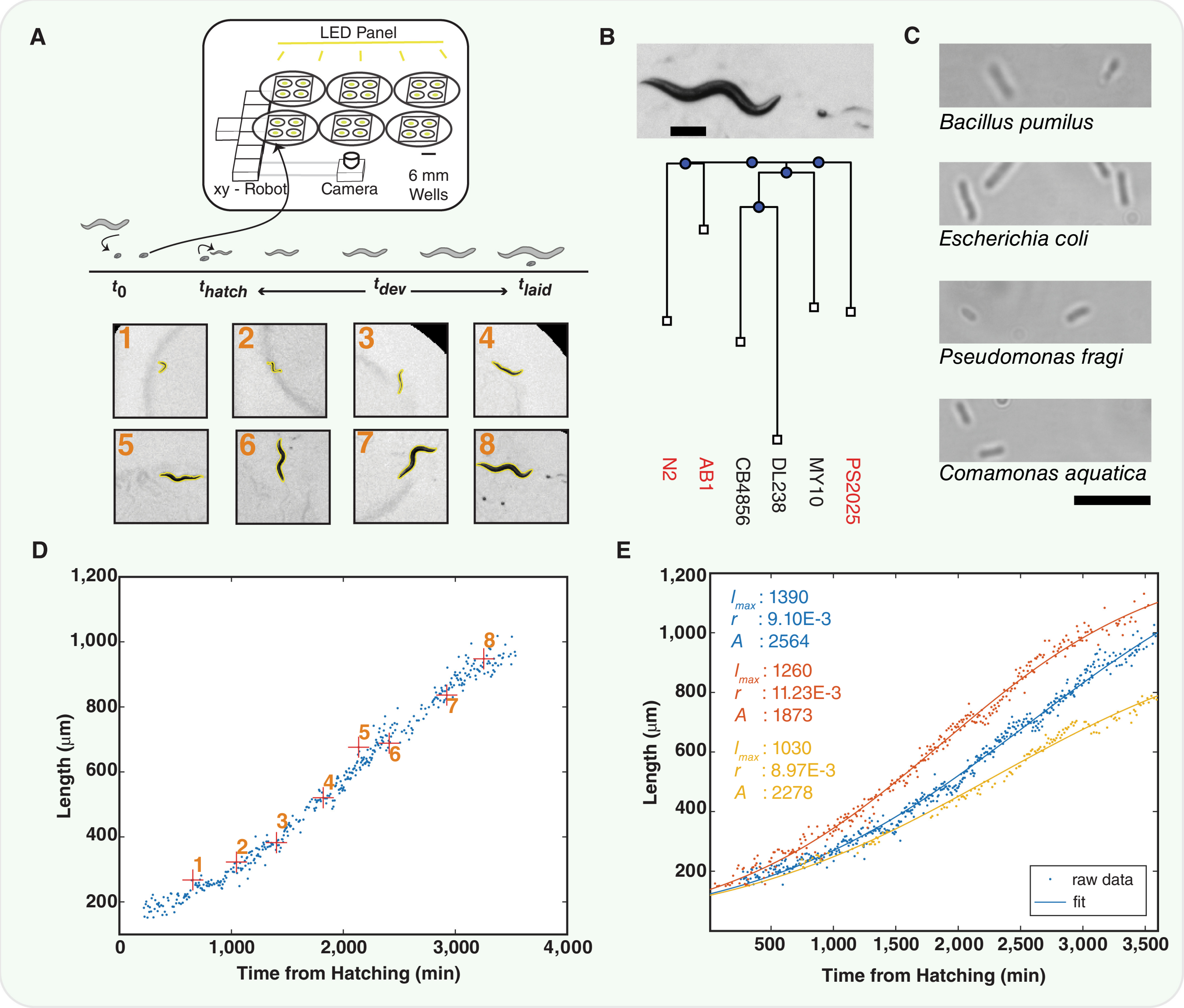


**Figure 1. Overview of the experimental apparatus, protocol and analysis.**

A A schematic of the imaging apparatus. Synchronised eggs are transferred into the mini wells at $t_0$. Hatching $t_{hatch}$ and egg-laying time $t_{laid}$ are manually recorded and give development time $t_{dev}$. Example images are shown for eight time points (A, lower), with the computed outline of the worm shown (yellow).

B An example image of an adult *C. elegans* and an egg (scale bar: 200 μm). A phylogenetic tree pruned from the CaeNDR (formerly CeNDR) database full tree. This shows the relationship of some common wild isolates (CB4856 is the Hawaiian strain) to the three strains used in this work, marked in red.

C Micro-graphs of the four bacteria used as food sources imaged at 100× magnification (scale bar: 10 μm).

D The entire developmental time course of an animal from hatching through adulthood. The blue points show calculated length over time, with eight specific points highlighted (red plus) corresponding to each of the images in (A, lower).

E Shows the length measurements and the computed logistic best-fit curves from three example time courses. The parameters of each logistic fit are shown to the left, with each colour corresponding to the relevant data and curve.

Source data are available online for this figure.

reflect the strong anticorrelation between the parameters $l_{max}$ and $r$. For positive values of $\phi_1$, animals grow quickly during their maximum growth phase but are ultimately shorter (Fig 4F, blue curve). In contrast, for negative $\phi_1$, animals grow more slowly at their peak, but are longer as adults (Fig 4F green curve). This may indicate a potential trade-off between the speed of growth during development and the ultimate size achieved by adult animals. In contrast, variation along $\phi_2$ results in both slower growth and shorter animals as adults (Fig 4G, green curve) or both faster and longer (Fig 4G, blue curve). Interestingly, variation along $\phi_1$ does not appear to affect the duration of reproductive development as much as along $\phi_2$ as shown by the colour of the plotted points (Fig 4D). Points away

 © 2023 The Authors

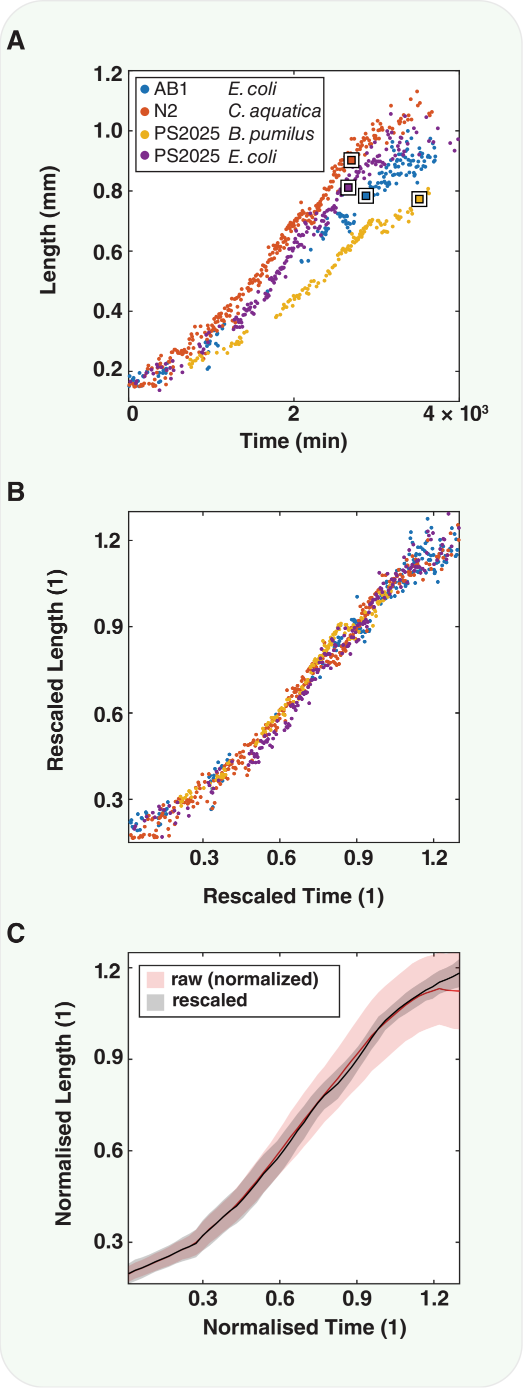


**Figure 2. Canalisation of developmentally rescaled growth curves.**

A Developmental time course of selected individuals where the square indicates $t_{dev}$ and $l_{dev}$ for each.

B The data can be rescaled to plot length $l$ as a fraction of development length $\hat{l} = l/l_{dev}$ and time $t$ as a fraction of development time $\hat{t} = t/t_{dev}$. Rescaling the data in this way changes the fit parameters of each logistic function. $[l_{max}, r, A] \rightarrow [l_{max}/l_{dev}, r \cdot t_{dev}, A/t_{dev}]$.

C The rescaling of the curves in B appears to collapse the growth curves. Growth curves rescaled by their individual $t_{dev}$ and $l_{dev}$ show a smaller standard deviation (shaded region = 1 SD) for the rescaled data (black) than for the raw data (red) (which is normalised to the average $t_{dev}$ and $l_{dev}$ to facilitate plotting on the same scale). Solid lines show the mean growth curve.

Source data are available online for this figure.

from the boundary in the positive $\phi_2$ direction correspond to slower reproductive development.

In addition to natural genetic variation and diet perturbations, the development of two *C. elegans* mutants was also measured. Because these mutants were not tested on the entire collection of diets, we chose not to include them in the analysis presented in Fig 4. The first mutant is *sid-2*, a deletion in a protein that is required for RNAi by feeding. This mutant has a mild developmental phenotype and hatches a few per cent longer than N2 and maintains this extra length through adulthood (Braukmann *et al*, 2021) with a faster mean development time (47.74 h). The second, *C28H8.3*, is a deletion in an uncharacterised gene that is orthologous to several human DExD/H-Box helicases, including DDX60. This deletion has a more severe developmental phenotype, increasing the mean development time by about 4.5 h (≈9%) compared with N2 (50.83 h) fed the same diet. The mild *sid-2* mutant lies very close to *wild-type* N2 (Fig 5C) and its distribution largely overlaps the marginal distributions for both its diet and the N2 genotype (Fig 5A and B, cyan curves). In contrast, the more severe mutant breaks both trends, clustering as an outlier to other *wild-type* N2 worms, and as an outlier to other worm strains fed on *E. coli* (Fig 5A and B, purple curves). However, the two mutants do cluster together along the third nonlinear principal component (Fig 5D, red curve). If we consider the 2d manifold defined by the first two nonlinear principal components, then mutations do indeed seem to drive development away from this manifold in the positive direction (Fig 5D and E). Interestingly, natural genetic variation also drives development off the manifold but in the opposite direction (Fig 5D, green curve) and only in combination with diet perturbations (Fig 5E). It is also interesting to note that the *sid-2* mutant is off-manifold in the same way as the C28 mutant despite it having a mild phenotype and not breaking diet and genotype trends.

## Discussion

Biological systems are remarkable in part because they are both incredibly robust and simultaneously flexible. Biological processes faithfully regenerate complex developmental programmes every generation, yet these same processes have given rise to an overwhelming diversity of complex matter. Canalisation and developmental plasticity appear to be opposing forces. One can imagine that these forces ebb and flow, acting in sequence. Cryptic variation, that is, genetic variation that does not result in phenotypic differences under normal conditions, but can be revealed in certain circumstances, for example, by the chaperone HSP-90 (Rutherford & Lindquist, 1998), is a good example of this, and it has been shown that disruption of the chaperone at the molecular level acts as the switch from robustness to plasticity, even in complex phenotypes such as the morphology of an animal (Sieriebriennikov & Sommer, 2018). However, this may be only part of the story. Canalisation and flexibility may in fact be complementary properties of

© 2023 The Authors

**Box 1. Illustration of relationships between genetic architecture, dimensionality and the correlation structure of traits**

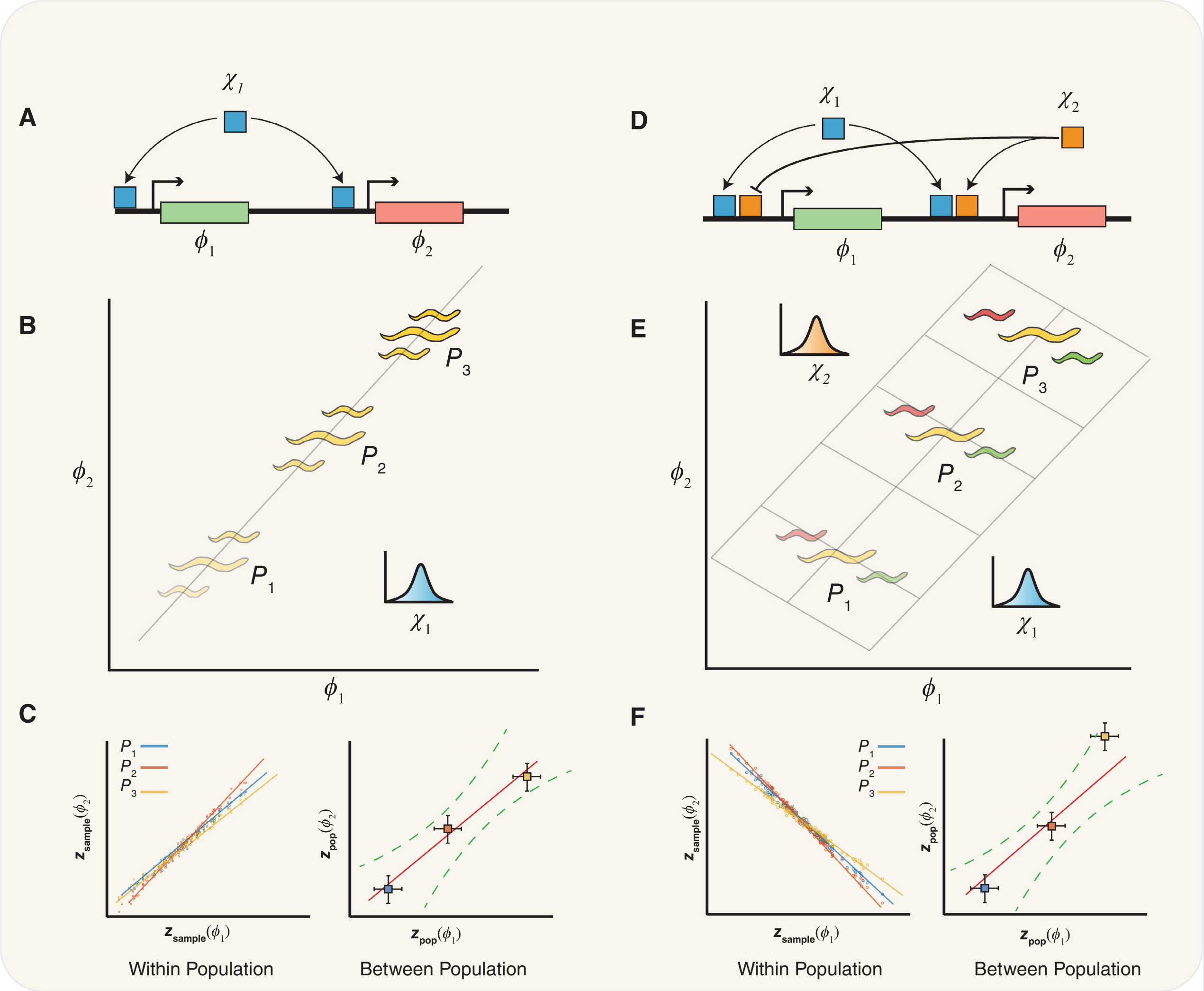


A A simple genetic circuit where green $\phi_1$ and red $\phi_2$ phenotypes are controlled by a single transcription factor $\chi_1$. The imaginary gene products produce red colour and green colour, which combines to produce yellow.

B Different populations ($P_1$–$P_3$) have different mean expression of $\chi_1$ due to either genetic or environmental changes, changing the brightness of the yellow colour. Fluctuations in $\chi_1$ around these mean values lead to correlated noise within populations.

C The mean values of $\phi_1$ and $\phi_2$ are also correlated between populations (right panel) in the same way. This leads to a one-dimensional phenotypic manifold (B, grey line), which is consistent with the single knob $\chi_1$.

D Here, there are two independent transcription factors ($\chi_1,\chi_2$) that control $\phi_1$ and $\phi_2$. $\chi_1$ controls both outputs in a correlated manner similarly to (A); however, the second factor $\chi_2$ activates $\phi_2$ but represses $\phi_1$.

E In each population, $P_1$–$P_3$ E, $\chi_1$ sets the mean value of both $\phi_1$ and $\phi_2$ in a correlated manner, changing the overall brightness of the yellow colour.

F Fluctuations in $\chi_2$ introduce anticorrelated noise within populations (left panel), shifting the phenotype to either green or red. The between-population correlation is dominated by the changes in $\chi_1$, resulting in correlated changes in both $\phi_1$ and $\phi_2$ (right panel). The within-population anticorrelation expands the dimensionality of the manifold (E, grey plane). Dimensionality can also be expanded by uncorrelated noise both within and between populations (not illustrated).

systems that are organised to generate low-dimensional phenotypic manifolds. Concentration of dimensionality is an intrinsic property of complex high-dimensional systems, even if only trivially because of the Johnson–Lindenstrauss lemma (Johnson & Lindenstrauss, 1984). However, the emergence of low dimensions in biological systems is often orders of magnitude smaller than random

 © 2023 The Authors

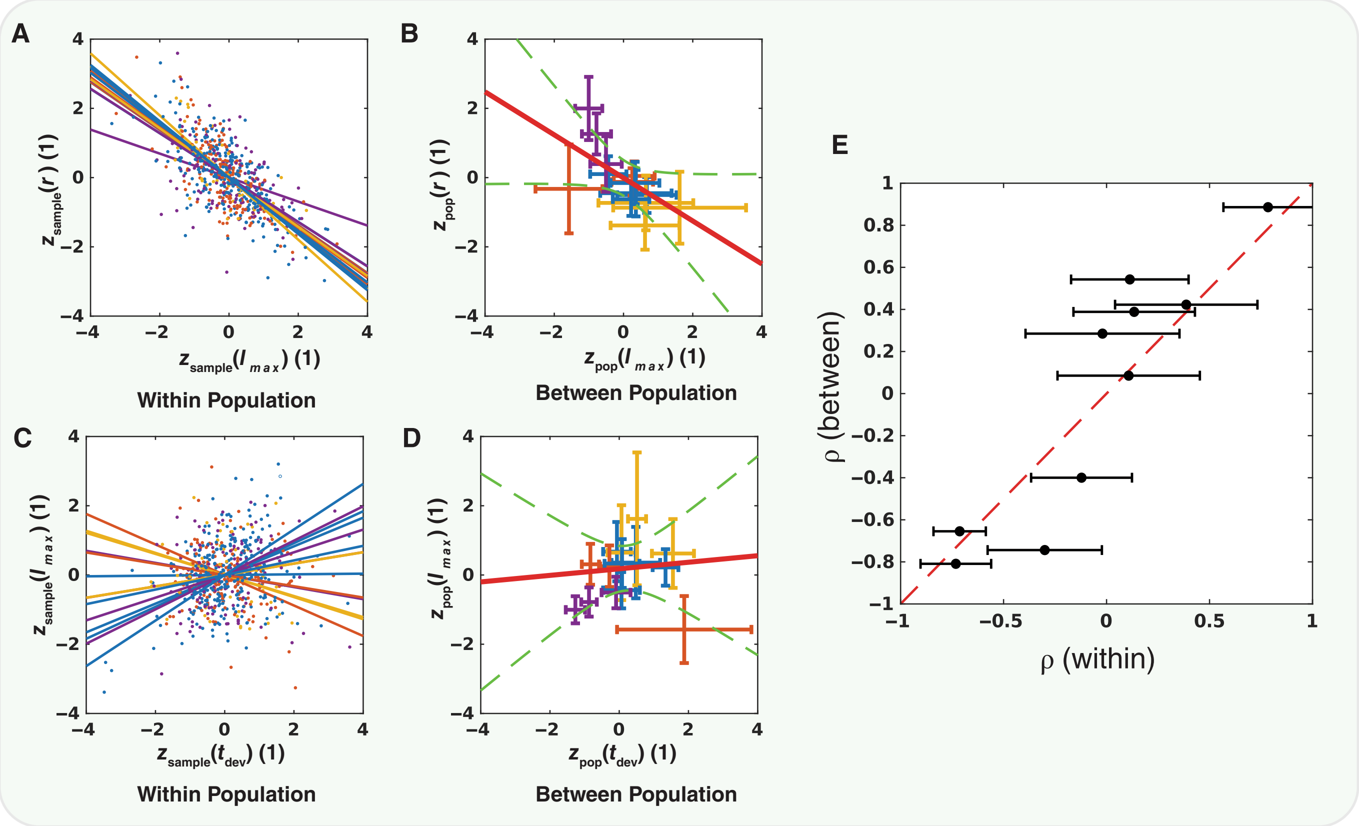


**Figure 3. Within-population correlations predict between-population correlations.**

A An example showing a pair of developmental parameters *l* and *r* that are correlated within each populations measured. The scatter plot shows z score of these parameters with respect to each conditions mean and variance, along with the best-fit linear regression for that condition (colours indicate the food source). There is a significant within-population negative correlation of these parameters in each condition.

B The z score is also calculated with respect to the mean and variance of all conditions taken together. These are then grouped by condition, and the mean and standard deviation of the z scores of both parameters are plotted (colours indicate the food source). The means are also correlated between conditions, as indicated by the confidence interval (green dashed lines) of the slope of the linear regression (red line). ($N$ = {64, 81, 22, 53, 79, 75, 13, 23, 64, 37, 24, 52, 64, 22} biological replicates).

C For other pairs of traits, there is no significant within-population correlation between traits, for example, between the development time $t_{dev}$ and *l*.

D Similarly, the between-population means are also not significantly correlated for this pair of traits. The means are plotted with their standard deviations. ($N$ = {64, 81, 22, 53, 79, 75, 13, 23, 64, 37, 24, 52, 64, 22} biological replicates).

E A summary of the within- and between-population correlation coefficient for all pairs or developmental parameters, the mean correlation coefficient for each of the within condition groups is plotted with error bars showing the standard deviation. For the correlation among the mean values, there is only a single value; thus, there are no vertical error bars. The red dashed line indicates equivalence, showing that the within-population correlations are largely but not exclusively predictive of the between-population correlations. ($N$ = 673 biological replicates for each trait pair).

Source data are available online for this figure.

projection would predict (Eckmann & Tlusty, 2021). At the heart of concentration of dimensionality lies projection. We propose that it is natural to view robustness and plasticity as analogous to projection of variation onto, or orthogonal to, a low-dimensional manifold, respectively. Given a distribution of variations that arise from environmental heterogeneity, stochastic fluctuations and genetic mutations, whether a given phenotype or set of phenotypes is buffered or responsive will depend on the projection function from high-dimensional chemical space to the low-dimensional phenotype space. In cases where a phenotype is highly buffered to some set of variations, almost all those variations will be orthogonal to the phenotypic manifold, and variations for which the phenotype is plastic will have large projections onto the manifold (Appendix Fig S3). In this scenario, one might envision the process of evolution as one of shaping the projection function, and thus the resulting phenotypic manifold, given the statistics of distribution of variations. Adaptation can then be viewed as the process of learning the optimal projection over the prior distribution of fluctuations, that is, an environment to phenotype rather than genotype to phenotype map (Xue *et al*, 2019), although the environment to phenotype map is certainly a product of the architecture of the gene regulatory networks of the organism.

It is tempting to look for a genetic basis for the emergence of low-dimensional phenotypes, especially in *C. elegans*, which has many available genetic tools. However, we believe that it is unlikely that a single-gene or a genetic module will underlie this process. Rather, it will likely be a property of the entire network of molecular interactions that underlie complex phenotypes. Nevertheless, there

© 2023 The Authors

may be important connections between the structure of phenotypic manifolds and evolutionary dynamics. Movement along, rather than away from such manifolds, has been proposed to constitute a path of "least resistance" for genetic changes that could fix phenotypic variations. While some evidence supports this hypothesis, for example, a study of the integration of phenotypic and life-history traits in the flowering plant *Arabidopsis thaliana* (Pigliucci & Hayden, 2001), other evidence from the morphospace of the greenfinch *Carduelis chloris* suggests that within-population correlations may not predict between-population correlations (Merilä & Björklund, 1999). This discrepancy may be due in part to the way traits are quantified, how the dimensionality reduction is performed and to whether the populations included in the analysis sample natural genetic variation, genetic mutations, environmental variations or combinations of all three. While the question of when and under what conditions the "directions" of phenotypic plasticity are predictive of the directions of subsequent genetic evolution remains open, phenotypic plasticity in canalised traits has been shown to be associated with rapid evolutionary diversification. An example comes from the polyphenism in the feeding structures of nematodes, in which the acquisition of mouth-form plasticity is associated with an increase in evolutionary rates. Interestingly, even if only one of the two alternative forms is fixed subsequently, the underlying genetic architecture seems to maintain an expanded phenotypic manifold that facilitates

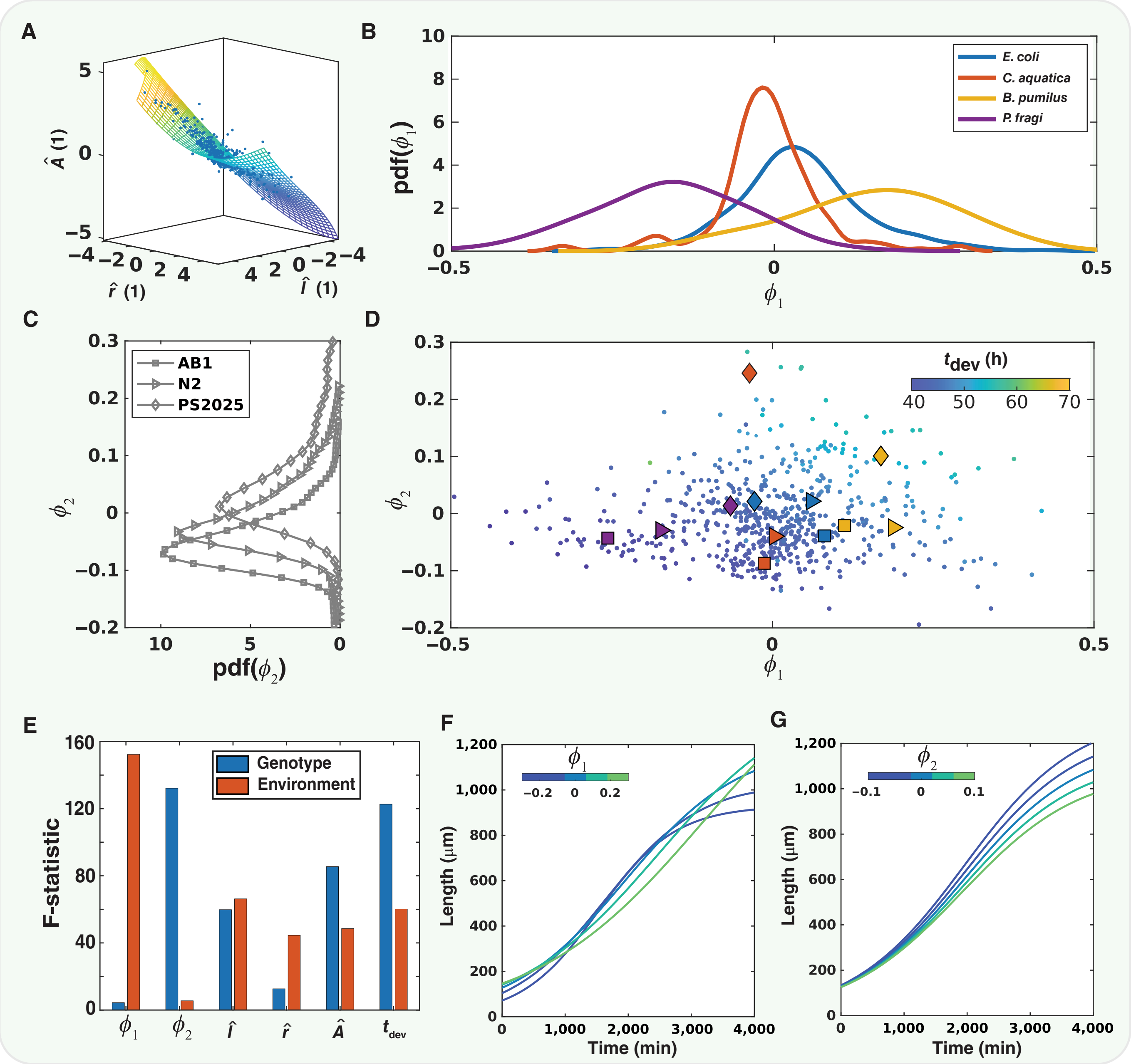


**Figure 4.**


© 2023 The Authors

◀ **Figure 4. Dimensionality reduction of developmental space using nonlinear principal component analysis.**

A The z scores of the three rescaled logistic fit parameters are shown in a 3d scatterplot (blue dots). These points lie close to a curved 2d manifold (mesh grid), which was found by performing nonlinear principal component analysis (NLPCA). The flattened manifold is shown in (D).

B On this manifold, $\phi_1$ seems to separate the environmental conditions as shown in by the marginal distribution over bacterial food sources.

C In contrast, the marginal distributions over the *C. elegans* strains shows separation in $\phi_2$. Marginal distributions were computed with a kernel density estimator implemented with the ksdensity function in MATLAB.

D The principal component weights for first two nonlinear principal components $\phi_1$ and $\phi_2$ for each *C. elegans* shown as a scatterplot. The colour of each point indicates that worm's development time, $t_{dev}$, as indicated by the colour bar. The mean $\phi_1$ and $\phi_2$ for each condition are shown as a combination of symbols (*C. elegans* strain) and colour (Bacterial food source).

E This separation in $\phi_1$ and $\phi_2$ can be quantified by computing the F-statistic for a linear regression model taking genotype and environment as regressors. $\phi_1$ regresses primarily on environment and $\phi_2$ on genotype. Interestingly, both genotype and environment together are generally required to explain the variance in the three logistic fit parameters and the developmental time alone.

F To determine the effect of varying $\phi_1$ on the shape of the growth curve, $\phi_2$ was fixed to 0 and $\phi_1$ was varied through a range, as indicated by the colour bar, with coordinates being converted back from the unit-less quantities. In this case, there appears to be a trade-off between fast growth (blue curves) and larger adult size (green curves).

G Similarly, when $\phi_1$ is fixed and $\phi_2$ varied, the resulting growth curves change from slower growth and smaller adult size (green) to faster growth and larger adult size (blue).

Source data are available online for this figure.

future exploration (Susoy *et al*, 2015). Although traditional forward and reverse genetics may not be the ideal approach, artificial evolution for expanded phenotypic manifolds seems promising, especially with an automated system that can map individuals to the phenotypic manifold in real time and use this as a selection criterion.

In physics, statistical mechanics provides tools to connect microscopic and macroscopic dynamics and to describe the behaviour of the macroscopic observables that arise from systems with large numbers of identical interacting components. Examples include the Navier–Stokes equation for fluid flow or the Fokker-Planck equation for diffusion. The coarse graining of many interacting degrees of freedom into a few dominant modes can be formulated precisely in terms of projection operators (Nakajima, 1958; Zwanzig, 1961; Mori, 1965). These projections often make use of time scale separations. For example, the random motion of a Brownian particle results from its many collisions with surrounding molecules, but these collisions occur very fast and can thus be treated as white noise. For near equilibrium systems, the rigorous relation between the fluctuations arising from the many unobserved degrees of freedom and the evolution of the system's macroscopic observables are known collectively as fluctuation–dissipation relations (Callen & Welton, 1951; Kubo, 1966). While biological systems are characteristically far from equilibrium, similar relations have been observed between phenotypic variability and evolutionary response (Sato *et al*, 2003; Tang & Kaneko, 2021). In fact, even in systems far from equilibrium, relations of this sort can arise similarly when there is a separation of both observed and unobserved degrees of freedom and of timescales (Jung & Schmid, 2021). While the origin of low-dimensional dynamics and of fluctuation–dissipation-like relations in biological systems has not been completely solved, some intriguing mechanisms have recently been proposed. For example, Furusawa & Kaneko (2018) propose that the evolution of robustness may be the origin of fluctuation–dissipation relations and of the emergence of low dimensionality in biological systems. They propose that if the same evolutionary forces that increase robustness to noise also tend to increase robustness to genetic perturbations, this could account for both phenomena (Furusawa & Kaneko, 2015; Kaneko & Furusawa, 2021). Recently, Murugan and colleagues have presented a model describing how mutational perturbations may be constrained by global epistasis to excite only a few slow or *soft* modes with examples from protein elasticity and gene regulatory dynamics (Husain & Murugan, 2020). Their work shows that such a

**Figure 5. Analysis of genetic mutations in relation to natural genetic variation and diet perturbations.** ▶

A As in Fig 4B, marginal distributions over each bacterial diet, however, this panel includes the two mutant *C. elegans* strains, sid-2 and C28H8.3, which were only tested on *E. coli*. Distributions are estimated using a kernel density estimator. The sid-2 mutant has a mild developmental phenotype and its distribution (cyan curve) is similar to the other *E. coli* fed animals. However, the more severe mutant, C28H8.3 (magenta curve), breaks this pattern and does not cluster together with the other *E. coli* fed animals.

B Likewise, as in Fig 4C, marginal distributions estimated with a kernel density estimator for each genotype, including the two mutants. The sid-2 mutant (cyan curve) is similar to the other strain from which it was derived from N2 (grey triangles). However, the C28H8.3 mutant (magenta curve) is distinct from N2 and from the other nonmutant strains in general (grey curves).

C Like Fig 4D, the nonlinear principal component embedding projected onto the first two nonlinear components $\phi_1$ and $\phi_2$ and including the mean for the two mutant strains, sid-2 (cyan circle) and C28H8.3, (magenta triangle). The sid-2 mutant is closer than C28H8.3 to N2, from which both were derived (blue triangle), and they fall on opposite sides on N2 in this plane.

D If we group the genotypes with wild-type, including N2 (blue), natural variation, including AB1 and PS2025 (green), and mutants, including sid-2 and C28H8.3 (red), these cluster along the third nonlinear principal component $\phi_3$. If we define the manifold as the plane spanned by $\phi_1$ and $\phi_2$ (blue line), then mutation drives development off the manifold. Natural genetic variation also does this, but these have opposite effects, with mutation increasing $\phi_3$ and natural variation decreasing it.

E A scatter plot of the embedding projected onto the first and third nonlinear principal components. The mean embedding is shown for each combination of diet and genotype. The two mutants lie above the manifold (blue line), and a few of the wild isolates lie below, but this only occurs in combination with a diet perturbation (yellow square, orange and yellow diamonds). The symbols are the same as in (C).

Source data are available online for this figure.

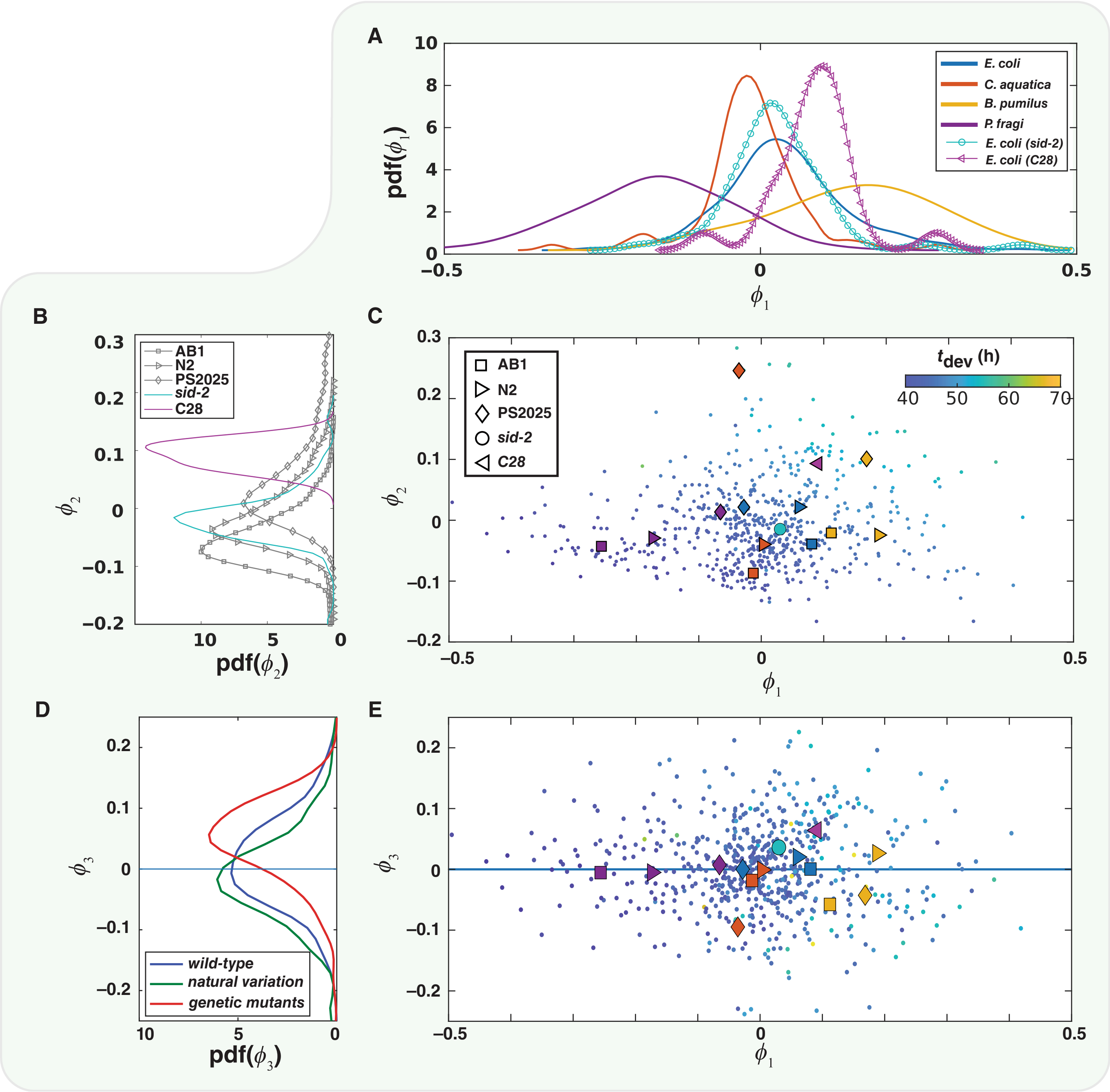


**Figure 5.**

relationship between mutational induced and physically induced deformations is expected mathematically for protein elasticity. The existence of such slow modes may also be related to the seemingly universal emergence of *stiff* and *sloppy* modes from parameter space compression (Gutenkunst *et al*, 2007; Machta *et al*, 2013).

In *C. elegans*, development has been shown to be controlled by a massive gene expression oscillator that is comprised of ≈3,700 genes (Hendriks *et al*, 2014; Meeuse *et al*, 2020). The existence of such an oscillator evokes a direct analogy to projection operator techniques as this network only has a few excitable oscillatory modes similar to a Fourier transform or to the cosine transform employed in Box 2. Recently, the components of a gene regulatory network that comprises a central clock to control this oscillator have been identified (Meeuse *et al*, 2023). Interestingly, Matsushita & Kaneko (2020) have shown how epigenetic modulation of oscillations can give rise to homeorhesis. As a follow-up to this work, we would like to assess how mutations in these core components affect how fluctuations in gene expression are projected onto the main oscillatory modes that control moulting, and how this in turn manifests on the developmental manifold of *C. elegans*.

 © 2023 The Authors

**Box 2. Illustration of robustness and flexibility resulting from projection onto a low-dimensional manifold.**

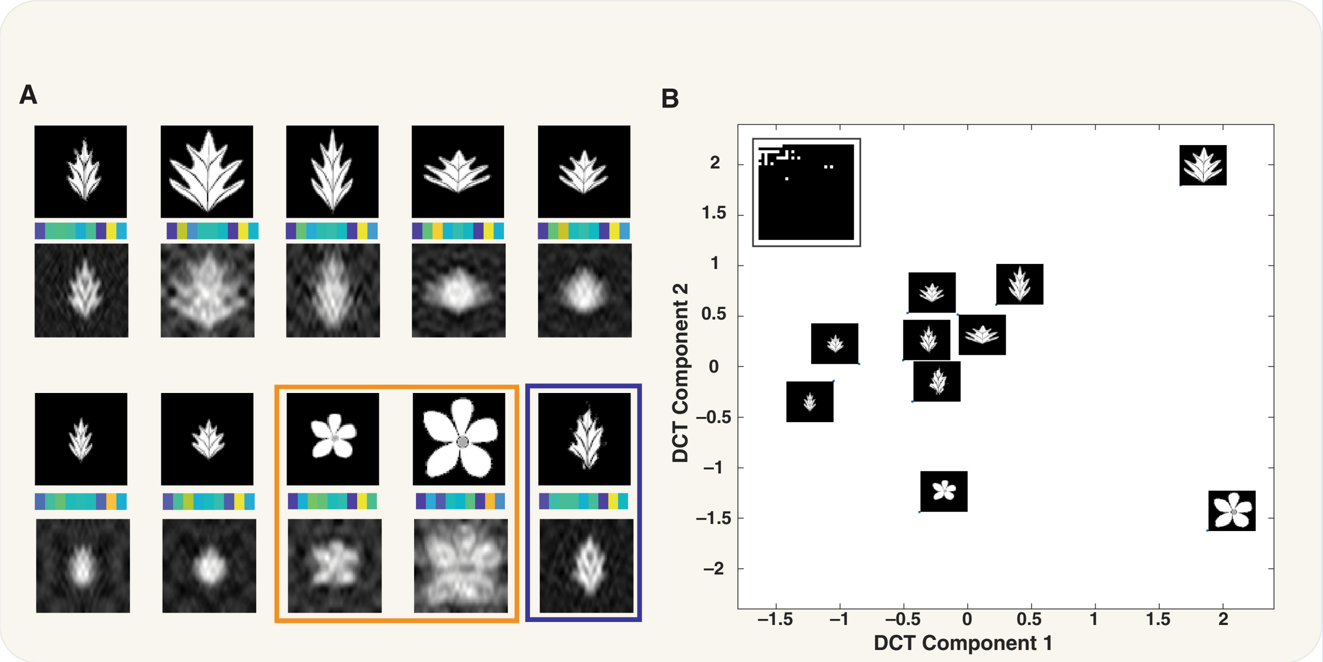


A In this example, 64 × 64 pixel leaf images represent a high-dimensional environment to be sensed. The 2d discrete cosine transform (DCT) is then used as a projection operator and implemented by the *dct2* function in MATLAB. Past evolutionary history has optimised this projection function to recognise the top left leaf in panel (A), and for this, the top 35 modes capture 90% of the energy. The retained modes are shown in (B, inset). In (A), we can see a variety of environmental inputs and their reconstructions from their low-dimensional representations; the first 10 mode weightings are shown as colour band below each image in (A). Although this projection operator evolved for the leaf in the top left, it reconstructs the different leaf images reasonably well. High-frequency variation around the original leaf shape is buffered (A, blue box), resulting in canalisation of the representation such fluctuations in the environmental input. Furthermore, even distinct environmental inputs such as the flowers (A, orange box) are reconstructed fairly well, demonstrating that this projection operator is robust, even for divergent inputs.

B This shows the embedding onto the first two DCT components. From this we can visualise both the robustness and the plasticity associated with this projection operator. The leaves cluster separately from the flowers, revealing flexibility, and the two leaves that only differ in high-frequency modes cluster together, showing robustness, with the variation between reconstructed representations smaller than that of inputs. Here, the first component is correlated with overall size and the second seems to separate leaves from flowers. It is interesting to note that the projection operator was not optimised in any way to distinguish leaves from flowers, and in fact, the eigenfunctions used are the generic ones from the DCT. Over evolutionary time, organisms could not only optimise which modes are retained to better capture the prior distribution over contexts but also could optimise the shape of the eigenfunctions themselves. This panel depicts an environmental sensing mechanism, but equally important, though not shown here, would be the phenotypic execution mechanism. The structure of the network that maps the low-dimensional representation of the environment back into a high-dimensional phenotype will also be an important source of variability and affect both robustness and plasticity.

In this work, we have focussed on variability resulting from combinations of dietary and genetic perturbations, and we have done so in only fixed environments. In the future, it will be interesting to perform experiments in which developmental trajectories are perturbed by some impulse or step function and to observe the resulting relaxation. *C. elegans* development is highly temperature dependent, and it would be interesting to first assay developmental trajectories at different temperatures and then to perturb development using various temperature shift protocols. In addition, if we could find perturbations that tend to generate displacement in specific directions on the phenotypic manifold, we could test quantitatively for fluctuation response type relationships. The projection operator perspective naturally leads one to consider how organisms might learn their particular projection function. Evolution by mutation and selection will surely play a large part in this process, especially when an organism must adapt to changes to the prior distribution of variations, but there is no reason, in principle, that this operator cannot also be shaped by within generation "learning" and other nongenetic and epigenetic feedback mechanisms.

Biological systems are remarkably robust, and yet the same mechanisms that underlie this robustness have also generated the incredible diversity of life on Earth. In this work, we attempt to reconcile these seemingly opposing forces by studying the structure of variability in the development of an animal in the contexts of different genetic backgrounds and environments. We have used high-resolution imaging data of the growth of the nematode *C. elegans* in environmental and genetic contexts to map the variability of development. We find that traits that are correlated within a particular

© 2023 The Authors 

context predict whether the mean values of those traits will be correlated among different contexts. Correlation between traits indicates that the true dimensionality of the system may be lower, and we find a parsimonious low-dimensional representation of the variability, in which the contributions of genetic variation on the phenotype are separable from those of environmental variation by a simple linear model. We present a framework in which the emergence of low dimensionality provides a basis for both robustness and plasticity. Concentration of dimensionality seems to be an intrinsic property of the chemical and molecular networks that generate living systems and of complex dynamical systems in general. While beyond the scope of this work, we hope that the connection between projection operators in physical systems, which explain how reproducible coarse-grained dynamics arise from the stochastic influences of innumerable microscopic components, may inform the theory in biology of how reproducible developmental dynamics arise from the interactions of similarly large numbers of biomolecules.

# Materials and Methods

### Reagents and Tools table

| Reagent/Resource | Reference or Source | Identifier or Catalogue Number |
|---|---|---|
| **Experimental Models** | | |
| *C. elegans* N2 | CGC https://cgc.umn.edu | N2 |
| *C. elegans* AB1 | CGC https://cgc.umn.edu | AB1 |
| *C. elegans* PS2025 | CGC https://cgc.umn.edu | PS2025 |
| *C. elegans sid2* (mj465) III | Fabian Braukmann EM Lab | SX3237 |
| *C. elegans c28h8.3* (mj649) III | Alexandra Dallaire EM Lab | SX3614 |
| *Comamonas aquatica* DA1877 | CGC https://cgc.umn.edu | DA1877 |
| *Escherichia coli* HB101 | CGC https://cgc.umn.edu | HB101 |
| *Bacillus pumilus* | Marie-Anne Felix Lab | JUb197-19 |
| *Pseudomonas fragi* | Marie-Anne Felix Lab | JUb239-19 |
| **Chemicals, Enzymes and other reagents** | | |
| Gelzan CM Gelrite | Merck/Sigma Life Sciences | Cat # G1910-250G, Lot # SLBL5174V |
| $KH_2PO_4$ | Merck/Sigma Aldrich | Cat # P9791 |
| NaCl | Merck/Sigma Aldrich | Cat # S3014 |
| $CaCl_2$ | Honeywell/Fluka | Cat # 223506 |
| $MgCl_2$ | Merck/Sigma Aldrich | Cat # M8266 |
| **Software** | | |
| MATLAB R2018b, R2022b | http://www.mathworks.com | License # 666637 |
| **Other** | | |
| 40 mm lens tube | Thor Labs | CML40 |
| 50 mm f/1.4 lens | Navitar | NMV-50 M1 |
| Eleksdraw Robot | Eleksmaker https://www.tomtop.com/p-e2350.html | Item#: E2350 |
| Flea3 3.2 MP monochrome camera (discontinued) | Teledyne FLIR | FL3-U3-32S2M-CS (discontinued) |
| Replacement for Flea3, Blackfly S USB3 Camera | Teledyne FLIR | BFS-U3-31S4M-C |
| A4 LED Light Panel | www.amazon.com | |
| Petroff-Hausser Counting Chamber | Hausser Scientific | Item# 3900 |
| LabJack U3 USB DAQ | LabJack | U3-LV |
| Linear Thermistor | Omega Engineering | Part # 44204 |
| Peltier Heater | Custom Thermoelectric | Part # 03111-5 M31-24CQ |
| NanoDrop One | Thermo Scientific | Cat # ND-ONE-W |


© 2023 The Authors

## Methods and Protocols

### Imaging hardware

The imaging hardware consists of a single 3.2 MP monochrome camera (Flea3, Teledyne FLIR) mounted to the arm of an XY-plotting robot (Eleksdraw, Eleksmaker) that moves the arm using two stepper motors controlled by a GRBL stepper motor controller that interprets the G code sent via a USB serial connection using the MATLAB (R2022b, Mathworks) *fprintf* command. Returning to the same position was accurate to with a few hundred microns, and wells were recentred periodically by fitting a circle to an intensity-threshold image of the well and zeroing the offset. Illumination was provided from above by an LED light panel. To maximise contrast, oblique illumination was blocked by a sheet of black acrylic in which an array of square holes was laser cut to allow direct illumination to pass though. A 50 mm f/1.4 lens (NMV-50M1, Navitar) was attached to the camera through a 40 mm lens tube (CML40, Thorlabs). In this system, one pixel in the image corresponded to 2.67 μm, giving an effective magnification of 0.93×. Detailed instructions and a supplier parts list are available on request.

### Multiwell plates

Multiwell plates were made by gently placing a pre-cut acrylic form into a standard 30 mm Falcon petri dish filled with 5 ml of liquefied NGM-Gelrite medium. The multiwell acrylic forms were 24 mm square, with a 2 × 2 grid of 6 mm diameter circular wells 4 cm from the edge and 10 cm centre to centre, and were cut from 3-mm-thick black acrylic using an LS 6090 PRO Laser Cutter (HPC Laser Ltd). After placing the multiwell plate into the molten solid media so that it rested on the surface, the plate was allowed to set and to dry for 1 h covered at 20°C and seeded with 2 μl of bacteria corresponding to approximately 18 million cells, as measured using a Petroff-Hausser Counting Chamber (Hausser Scientific).

### Temperature control

Plates were kept in an insulated, temperature-controlled box during imaging, which itself was in a temperature-controlled room set to 20°C. The actual average temperature in the room was 19.7°C. Temperatures were measured with a custom thermometer; the signal from a linear thermistor (Omega Engineering) was difference amplified against a known voltage corresponding to 20°C. The amplified signal (Gain = 2) was recorded in MATLAB (Mathworks) from the analogue input of a DAQ (Labjack). This was fed into a proportional–integral–differential (PID) control script whose output was a voltage that controlled a push-pull current amplifier driving a Peltier effect element (Custom Thermoelectric). Fans were used to distribute air from the heat sinks on each face of the Peltier element either into the enclosure or as exhaust. Temperature was maintained to within 70 mK of the set point (20°C).

### Image processing

Images were captured directly into MATLAB using the built-in video input object class. Moving objects were extracted from each image using background subtraction, to generate a region of interest with the largest amount of detected motion, as measured by the largest pixel difference. Within this region of interest, objects were detected by contrast with the background by applying a threshold to a Laplacian of Gaussian filtered image. Filtering was performed using the MATLAB *imfilter* function with the *fspecial* function with filter size 15 × 15 and filter standard deviation of 1.5. Connected components were extracted from this thresholded image, and the locations and properties of connected components were recorded from the resulting black and white image. Area (in pixels) and centroid location were calculated using the MATLAB function *regionprops*, and length was computed using the MATLAB function *bwmorph* to extract the skeleton and the function *bwgeodesic* to compute the length. These properties were used to classify each blob as a worm or not, based on the output of a pretrained support vector machine. All images were also saved for later inspection, which was used to determine the egg-hatching and egg-laying times manually. All code used for analysis and data processing is available on GitHub at https://github.com/davex0r/DevelopmentalManifold, including data from processed images (raw images are available on request).

### Nematode culture and strains

*Caenorhabditis elegans* was grown on NGM agar plates and fed *E. coli* HB101 for standard maintenance at 20°C. Bristol N2 was used as wild-type strain (Brenner, 1974). In addition to N2, two wild isolate strains were used, AB1 isolated in Adelaide, Australia and PS2025 isolated in Pasadena, California. In addition, two single-gene mutants of N2 were used, *sid-2* (mj465), a mutant that is not competent for RNAi by feeding, and *c28h8.3* (mj649) a catalytic mutant of a putative helicase.

### Bacterial culture and strains

In addition to *E. coli* HB101, three other bacteria were used as food sources. *Comamonas aquatica* DA1877 (Avery & Shtonda, 2003) was used as it has been previously shown to increase the rate of *C. elegans* development by providing supplemental vitamin B12 (MacNeil *et al*, 2013; Watson *et al*, 2014). We also chose *Bacillus pumilus* and *Pseudomonas fragi* from the wild bacteria collection (Frézal & Félix, 2015) as we had previously observed large developmental differences in these food sources. Optical density (OD) of bacterial cultures was measured on a NanoDrop spectrophotometer (Thermo Scientific).

### Synchronisation by coordinated egg laying

To generate populations of synchronised animals without bleaching and starvation, young egg-laying adults (50–75-h posthatching) were gently picked with a platinum wire to a fresh NGM plate seeded with the appropriate bacteria and allowed to lay eggs for a fixed duration, after which the adults were removed from the plate and the eggs were collected. The egg-laying rate of animals at this stage is ≈6 eggs/animal/h. Synchronisation could be tightened by shortening the duration of egg laying, and the number of synchronised eggs could be increased by using more egg-laying adults.

### Nematode growth media—Gelrite

NGM-Gelrite plates were made by replacing the Agar in normal NGM recipe with Gelzan Gellan Gum (Sigma Life Sciences), a polymer derived from algae. The recipe is given in Table 1. In addition, peptone and cholesterol were omitted to prevent bacterial growth on the plate, so that the only available bacterial food was that which was initially inoculated. In this way, there were areas on the plate both where food was present and absent (Appendix Fig S5).

© 2023 The Authors 

**Table 1. Recipe for NGM-Gelrite.**

| Ingredient | Final concentration |
|---|---|
| NaCl | 3.0 g/l |
| Gellan Gum | 8.0 g/l |
| *Autoclave together* | |
| $CaCl_2$ | 1 mM |
| $MgCl_2$ | 1 mM |
| $KH_2PO_4$ | 25 mM |
| *Add Aseptically* | |

Additive salts were prepared in 1 M stock solutions, and the $KH_2PO_4$ stock solution was adjusted to pH 6.

#### *Phylogenetic tree of wild isolates*

The phylogenetic tree of *C. elegans* wild isolates was generated from the full CaeNDR (formally CeNDR) phylogenetic tree (Cook *et al*, 2017) which was hard-filtered by isotype using the *prune* function in MATLAB.

#### *Logistic fits*

Logistic fits were calculated using the MATLAB implementation of the Levenberg Marquardt (Marquardt, 1963) nonlinear least squares algorithm within the *lsqcurvefit* package. Logistic fits were performed on the raw data as well as the rescaled data. The rescaled fits were nearly identical to the raw-fit parameters, which were transformed according to equation (2) (see Fig EV1).

#### *Nonlinear PCA*

Nonlinear principal component analysis attempts to find an optimal auto encoder that can recreate the input data after passing it through a bottleneck layer with fewer components than the input. The number of components in the bottleneck layer corresponds to the number of desired principal components (Appendix Fig S4). The NLPCA implementation we use is from NLPCA toolbox for MATLAB (Scholz *et al*, 2005) and uses a multilayer perceptron architecture with a hyperbolic tangent activation function in the hidden layers.

#### *Probability density function (PDF) estimation*

A probability density function (PDF) is a nonnegative Lebesgue integrable function $f_x$, which satisfies the equation:

$$\Pr[a < X < b] = \int_a^b f_x dx$$

That is, the probability that the random variable $X$ takes a value in the range $[a, b]$ is given as the integral of the PDF on the interval. The integral of the PDF over the entire range $[-\infty, \infty]$ is thus equal to 1. In this work, we estimate the PDF using a kernel density estimator. The estimated PDF $f_x^{est}$ is given by the convolution of a Kernel function with Kronecker delta functions at each of the observed data points. Here, we use Gaussian distributions as our Kernel function.

$$f_x^{est}(\mathrm{x}) = \frac{1}{nh}\sum_{i=1}^{n} K\left(\frac{x - x_i}{h}\right)$$

with:

$$K(x) = \frac{1}{2\pi} exp\left[-\frac{x^2}{2}\right]$$

The parameter $h$ is called the bandwidth and is optimally chosen on the basis of the number of data points $n$. We use the *ksdensity* implementation of this estimator in MATLAB.

#### *Linear regression*

Each phenotypic output can be decomposed as a genetic contribution, an environmental contribution and some residual error. Linear regression was performed using the *fitlm* function in MATLAB. if $y$ is the phenotypic variable of interest,

$$y(G, E) = \beta_0 + \sum_i \beta_{1,i} G + \sum_j \beta_{2,j} E + \in$$

were fit with $y \in \left(\phi_1, \phi_2, \hat{l}, \hat{r}, \hat{A}\right)$, $G$ and $E$ are indicator variables which take the value of 1 or 0 depending of the strain $i$ and food $j$ in that condition, and $\in$ are the residuals to be minimised. *fitlm* uses an iteratively reweighted least squares algorithm. For example, the best-fit linear model for $\phi_1$ indicates that the average value of $\phi_1$ is 0.022 and that changing to *Bacillus pumilus* increases $\phi_1$ by 0.115 while switching to *Pseudomonas fragi* moves $\phi_1$ by −0.200. F-statistics were calculated by the MATLAB *anova* function and are given by the formula:

$$F = \mathbb{V}[\mathbb{E}(y_i)]/\mathbb{E}[\mathbb{V}(y_i)]$$

where $\mathbb{V}(X)$ denotes the variance of the random variable $X$ and $\mathbb{E}(X)$ its expectation value. These are calculated for each prediction variable $y \in \left(\phi_1, \phi_2, \hat{l}, \hat{r}, \hat{A}\right)$ and decomposed according to environment or genotype $i \in G, E$.

#### *Measurement summary table*

| Variable name | Units | *n*/Worm (total) | Type | Description |
|---|---|---|---|---|
| Length ($l(t)$) | mm | ~200 (133,359) | Automatic | Time series of length |
| Ex-utero development ($t_{hatch}$) | s | 1 (673) | Manual | Duration from an egg being laid until it hatches |
| Length at hatching ($l_{hatch}$) | mm | 1 (673) | Manual | Length of the worm right after hatching |
| Reproductive development ($t_{dev}$) | s | 1 (673) | Manual | Duration from an egg being laid until the adult it becomes lays its first egg |
| Reproductive Length ($l_{dev}$) | mm | 1 (673) | Manual | Length of the adult worm when it lays its first egg |
| Logistic supremum $l_{max}$ | mm | 1 (673) | Fit | Fit from $l(t)$ time series |

 © 2023 The Authors

**Reagents and Tools table** (continued)

| Variable name | Units | *n*/Worm (total) | Type | Description |
|---|---|---|---|---|
| Logistic rate *r* | $s^{-1}$ | 1 (673) | Fit | Fit from $l(t)$ time series |
| Logistic midpoint *A* | s | 1 (673) | Fit | Fit from $l(t)$ time series |
| Rescaled supremum ($\hat{l}$) | [1] | 1 (673) | Derived | $\hat{l} = \frac{l_{max}}{l_{dev}}$ |
| Rescaled rate ($\hat{r}$) | [1] | 1 (673) | Derived | $\hat{r} = r \cdot t_{dev}$ |
| Rescaled midpoint (Â) | [1] | 1 (673) | Derived | $\hat{A} = \frac{A}{t_{dev}}$ |

***Experimental conditions summary table***

| | ***E. coli* HB101** | ***C. aquaticus* DA 1877** | ***B. pumilus*** | ***P. fragi*** |
|---|---|---|---|---|
| N2 (wild-type *C. elegans*) | *N* = 64 | *N* = 81 | *N* = 22 | *N* = 53 |
| AB1 (*C. elegans* natural genetic variation) | *N* = 79 | *N* = 75 | *N* = 13 | *N* = 23 |
| PS2025 (*C. elegans* natural genetic variation) | *N* = 64 | *N* = 37 | *N* = 24 | *N* = 52 |
| *sid-2* (*C. elegans* single-gene deletion, mild phenotype) | *N* = 64 | | | |
| C28H8.3 (*C. elegans* single-gene deletion, severe phenotype) | *N* = 22 | | | |

# Data availability

All essential data for the figures are available as Source Data. In addition, a complete repository is also available on GitHub at https://github.com/davex0r/DevelopmentalManifold.

**Expanded View** for this article is available online.

## Acknowledgements

This work was supported in part by grants to the Gurdon Institute (092096/Z/10/Z) and to EAM (104640/Z/14/Z) from the Wellcome Trust. In addition, DJJ was supported by a Herchel Smith postdoctoral fellowship. The authors would like to thank Marie-Anne Félix for providing us with wild isolates of *C. elegans* as well as wild bacteria. We would also like to thank Stanislas Leibler, Bing Kan Xue, Maroš Pleška, Jon Chuang and Ricardo Rao for helpful discussion, and in particular S.L. for advice on the presentation of the material and B.K.X. for suggesting the rescaling and the linear decomposition of the variance.

## Author contributions

**David J Jordan:** Conceptualization; resources; data curation; software; formal analysis; supervision; funding acquisition; validation; investigation; visualization; methodology; writing – original draft; project administration; writing – review and editing. **Eric A Miska:** Conceptualization; resources; supervision; funding acquisition; methodology; project administration; writing – review and editing.

## Disclosure and competing interests statement

The authors declare that they have no conflict of interest.

# References

Alba V, Carthew JE, Carthew RW, Mani M (2021) Global constraints within the developmental program of the *Drosophila* wing. *Elife* 10: e66750

Avery L, Shtonda BB (2003) Food transport in the *C. elegans* pharynx. *J Exp Biol* 206: 2441–2457

Bialek W (2022) On the dimensionality of behavior. *Proc Natl Acad Sci USA* 119: e2021860119

Braukmann F, Jordan D, Jenkins B, Koulman A, Miska EA (2021) SID-2 negatively regulates development likely independent of nutritional dsRNA uptake. *RNA Biol* 18: 888–899

Brennan C, Proekt A (2019) A quantitative model of conserved macroscopic dynamics predicts future motor commands. *Elife* 8: e46814

Brenner S (1974) The genetics of *Caenorhabditis elegans*. *Genetics* 77: 71–94

Callen HB, Welton TA (1951) Irreversibility and generalized noise. *Phys Rev* 83: 34–40

Campàs O, Mallarino R, Herrel A, Abzhanov A, Brenner MP (2010) Scaling and shear transformations capture beak shape variation in Darwin's finches. *Proc Natl Acad Sci USA* 107: 3356–3360

Chuang JS, Frentz Z, Leibler S (2019) Homeorhesis and ecological succession quantified in synthetic microbial ecosystems. *Proc Natl Acad Sci USA* 116: 14852–14861

Cook DE, Zdraljevic S, Roberts JP, Andersen EC (2017) CeNDR, the *Caenorhabditis elegans* natural diversity resource. *Nucleic Acids Res* 45: D650–D657

Costa AC, Ahamed T, Jordan D, Stephens GJ (2023) Maximally predictive states: From partial observations to long timescales. *Chaos* 33: 023136

Eckmann J-P, Tlusty T (2021) Dimensional reduction in complex living systems: Where, why, and how. *Bioessays* 43: e2100062

Filina O, Demirbas B, Haagmans R, van Zon JS (2022) Temporal scaling in *C. elegans* larval development. *Proc Natl Acad Sci USA* 119: e2123110119

Frentz Z, Kuehn S, Leibler S (2015) Strongly deterministic population dynamics in closed microbial communities. *Phys Rev X* 5: 041014

Frézal L, Félix M-A (2015) *C. elegans* outside the Petri dish. *Elife* 4: e05849

Furusawa C, Kaneko K (2015) Global relationships in fluctuation and response in adaptive evolution. *J R Soc Interface* 12: 20150482

Furusawa C, Kaneko K (2018) Formation of dominant mode by evolution in biological systems. *Phys Rev E* 97: 042410

Gutenkunst RN, Waterfall JJ, Casey FP, Brown KS, Myers CR, Sethna JP (2007) Universally sloppy parameter sensitivities in systems biology models. *PLoS Comput Biol* 3: 1871–1878

Hendriks G-J, Gaidatzis D, Aeschimann F, Großhans H (2014) Extensive oscillatory gene expression during *C. elegans* larval development. *Mol Cell* 53: 380–392

Huang S, Eichler G, Bar-Yam Y, Ingber DE (2005) Cell fates as high-dimensional attractor states of a complex gene regulatory network. *Phys Rev Lett* 94: 128701

Husain K, Murugan A (2020) Physical constraints on epistasis. *Mol Biol Evol* 37: 2865–2874

Johnson WB, Lindenstrauss J (1984) Extensions of Lipschitz mappings into a Hilbert space. In *Conference in Modern Analysis and Probability*, pp 189–206. Providence, RI: American Mathematical Society

Jordan D, Kuehn S, Katifori E, Leibler S (2013) Behavioral diversity in microbes and low-dimensional phenotypic spaces. *Proc Natl Acad Sci USA* 110: 14018–14023

Jung G, Schmid F (2021) Fluctuation-dissipation relations far from equilibrium: a case study. *Soft Matter* 17: 6413–6425

Kaneko K, Furusawa C (2021) Direction and constraint in phenotypic evolution: dimension reduction and global proportionality in phenotype fluctuation and responses. In *Evolutionary Systems Biology: Advances, Questions, and Opportunities*, Crombach A (ed), pp 35–58. Cham: Springer International Publishing

Klumpp S, Hwa T (2014) Bacterial growth: global effects on gene expression, growth feedback and proteome partition. *Curr Opin Biotechnol* 28: 96–102

Kubo R (1966) The fluctuation-dissipation theorem. *Rep Prog Phys* 29: 255

Machta BB, Chachra R, Transtrum MK, Sethna JP (2013) Parameter space compression underlies emergent theories and predictive models. *Science* 342: 604–607

MacNeil LT, Watson E, Arda HE, Zhu LJ, Walhout AJM (2013) Diet-induced developmental acceleration independent of TOR and insulin in *C. elegans*. *Cell* 153: 240–252

Marquardt DW (1963) An algorithm for least-squares estimation of nonlinear parameters. *J Soc Indust Appl Math* 11: 431–441

Matsushita Y, Kaneko K (2020) Homeorhesis in Waddington's landscape by epigenetic feedback regulation. *Phys Rev Res* 2: 023083

Meeuse MW, Hauser YP, Morales Moya LJ, Hendriks G-J, Eglinger J, Bogaarts G, Tsiairis C, Großhans H (2020) Developmental function and state transitions of a gene expression oscillator in *Caenorhabditis elegans*. *Mol Syst Biol* 16: e9498

Meeuse MWM, Hauser YP, Nahar S, Smith AAT, Braun K, Azzi C, Rempfler M, Großhans H (2023) *C. elegans* molting requires rhythmic accumulation of the Grainyhead/LSF transcription factor GRH-1. *EMBO J* 42: e111895

Merilä J, Björklund M (1999) Population divergence and morphometric integration in the greenfinch *Carduelis chloris* – evolution against the trajectory of least resistance? *J Evol Biol* 12: 103–112

Mori H (1965) Transport, collective motion, and Brownian motion. *Prog Theor Phys* 33: 423–455

Nakajima S (1958) On quantum theory of transport phenomena. *Prog Theor Phys* 20: 948–959

Pigliucci M (2003) Phenotypic integration: studying the ecology and evolution of complex phenotypes. *Ecol Lett* 6: 265–272

Pigliucci M, Hayden K (2001) Phenotypic plasticity is the major determinant of changes in phenotypic integration in *Arabidopsis*. *New Phytol* 152: 419–430

Pleška M, Jordan D, Frentz Z, Xue B, Leibler S (2021) Nongenetic individuality, changeability, and inheritance in bacterial behavior. *Proc Natl Acad Sci USA* 118: e2023322118

Prusinkiewicz P, Erasmus Y, Lane B, Harder LD, Coen E (2007) Evolution and development of inflorescence architectures. *Science* 316: 1452–1456

Raup DM (1966) Geometric analysis of shell coiling: general problems. *J Paleo* 40: 1178–1190

Rulands S, Simons B (2017) Emergence and universality in the regulation of stem cell fate. *Curr Opin Syst Biol* 5: 57–62

Rutherford SL, Lindquist S (1998) Hsp90 as a capacitor for morphological evolution. *Nature* 396: 336–342

Samuel BS, Rowedder H, Braendle C, Félix M-A, Ruvkun G (2016) *Caenorhabditis elegans* responses to bacteria from its natural habitats. *Proc Natl Acad Sci USA* 113: E3941–E3949

Sato K, Ito Y, Yomo T, Kaneko K (2003) On the relation between fluctuation and response in biological systems. *Proc Natl Acad Sci USA* 100: 14086–14090

Scholz M, Kaplan F, Guy CL, Kopka J, Selbig J (2005) Non-linear PCA: a missing data approach. *Bioinformatics* 21: 3887–3895

Shoval O, Sheftel H, Shinar G, Hart Y, Ramote O, Mayo A, Dekel E, Kavanagh K, Alon U (2012) Evolutionary trade-offs, pareto optimality, and the geometry of phenotype space. *Science* 336: 1157–1160

Sieriebriennikov B, Sommer RJ (2018) Developmental plasticity and robustness of a nematode mouth-form polyphenism. *Front Genet* 9: 382

Smith DJ, Lapedes AS, de Jong JC, Bestebroer TM, Rimmelzwaan GF, Osterhaus ADME, Fouchier RAM (2004) Mapping the antigenic and genetic evolution of influenza virus. *Science* 305: 371–376

Stephens G, Johnson-Kerner B, Bialek W, Ryu W (2008) Dimensionality and dynamics in the behavior of *C. elegans*. *PLoS Comput Biol* 4: e1000028

Stojanovski K, Großhans H, Towbin BD (2022) Coupling of growth rate and developmental tempo reduces body size heterogeneity in *C. elegans*. *Nat Commun* 13: 3132

Susoy V, Ragsdale EJ, Kanzaki N, Sommer RJ (2015) Rapid diversification associated with a macroevolutionary pulse of developmental plasticity. *Elife* 4: e05463

Tang Q-Y, Kaneko K (2021) Dynamics-evolution correspondence in protein structures. *Phys Rev Lett* 127: 098103

Turk M, Pentland A (1991) Eigenfaces for recognition. *J Cogn Neurosci* 3: 71–86

Waddington CH (1942) Canalization of development and the inheritance of acquired characters. *Nature* 150: 563–565

Waddington CH (1957) *The strategy of the genes: a discussion of some aspects of theoretical biology*. London, UK: George Allen & Unwin. https://wellcomecollection.org/works/nzwm3z65

Watson E, MacNeil LT, Ritter AD, Yilmaz LS, Rosebrock AP, Caudy AA, Walhout AJM (2014) Interspecies systems biology uncovers metabolites affecting *C. elegans* gene expression and life history traits. *Cell* 156: 759–770

Xue B, Sartori P, Leibler S (2019) Environment-to-phenotype mapping and adaptation strategies in varying environments. *Proc Natl Acad Sci USA* 116: 13847–13855

Zwanzig R (1961) Memory effects in irreversible thermodynamics. *Phys Rev* 124: 983–992


**License:** This is an open access article under the terms of the Creative Commons Attribution License, which permits use, distribution and reproduction in any medium, provided the original work is properly cited.



© 2023 The Authors

# Expanded View Figures

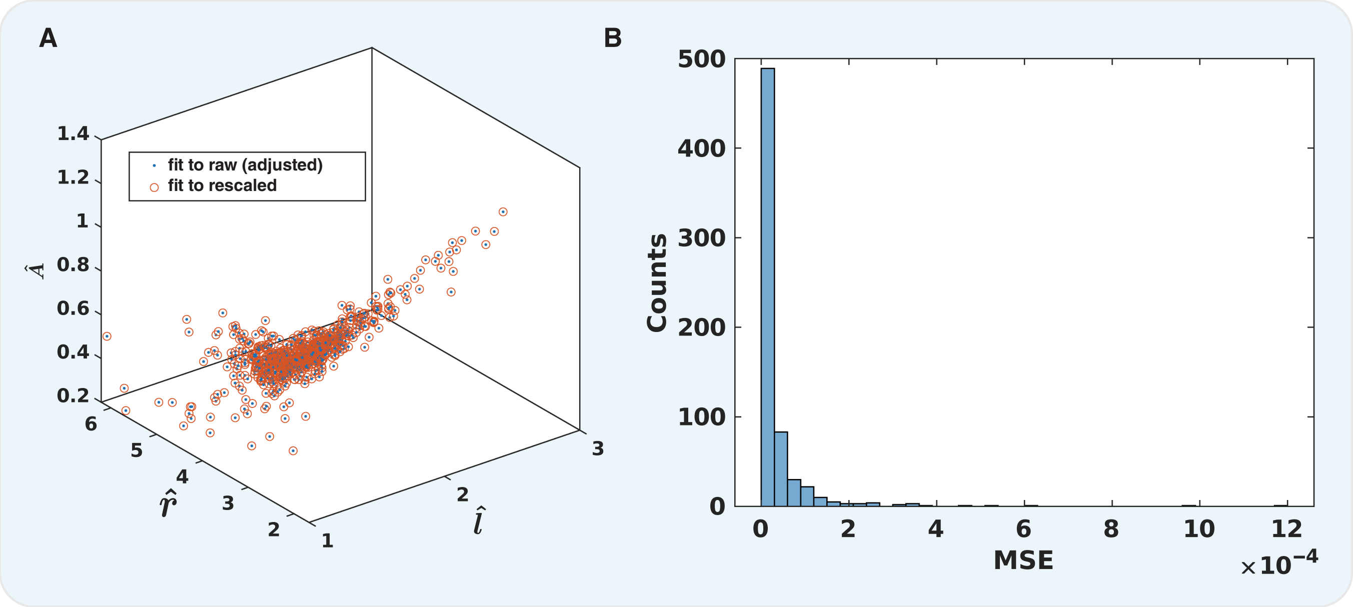


**Figure EV1. Rescaled logistic fit parameters are equivalent to fits performed on rescaled data.**

A Scatter plot of the three logistic fit parameters for each curve that were obtained either by fitting the raw data directly and renormalising the fit parameters with respect to the development time (blue points) or rescaling the data and then performing the fit (orange points).

B Histogram of the mean squared error between the raw adjusted and the rescaled fit data points.

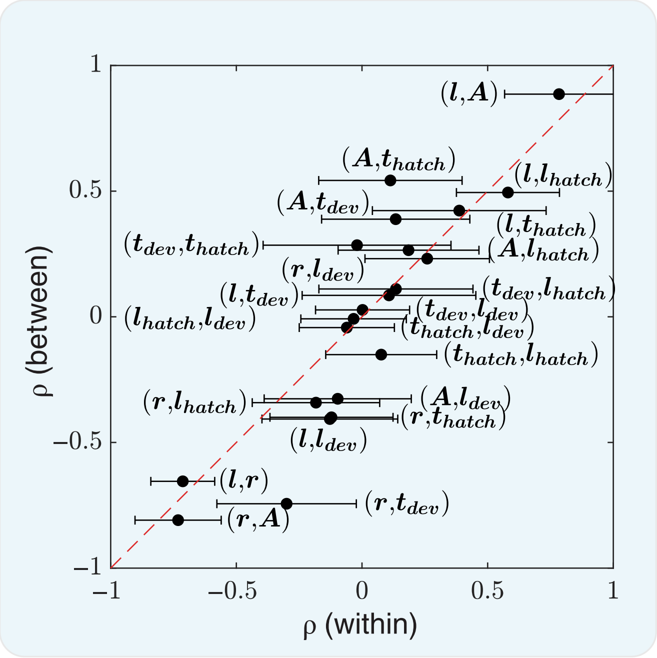


**Figure EV2. Detailed within-population vs. between-population trait pair correlations.**

Trait pairs within- vs. between-population correlations are plotted for an extended collection of traits (21 pairs of 7 traits) c.f. Fig 3E (10 pairs of 5 traits). The mean of the within-population correlation coefficients is plotted with the standard deviation as error bars against the between-population correlation coefficient. The red dashed line indicates equivalence. $N = 673$ biological replicates for each trait pair. The pairs of traits are annotated on the figure. In addition to ($l$, $r$, $A$, $t_{dev}$, $t_{hatch}$) shown in Fig 3E, this plot also includes $l_{hatch}$ the length of the newly hatched animal, and $l_{dev}$, the length of the adult animal when it lays its first egg.

© 2023 The Authors
