# Supplementary material for: Canalisation and plasticity on the developmental manifold of Caenorhabditis elegans
Source: Mol Syst Biol. 2023 Oct 18;19(11):e11835. doi: 10.15252/msb.202311835 (PMC10632735; doi:10.15252/msb.202311835)
Supplement: Supplementary file 9 [file MSB-19-e11835-s001.pdf]

Canalisation and plasticity on the developmental manifold of Caenorhabditis elegans

David Jordan and Eric Miska
DOI: 10.15252/msb.202311835

Corresponding author(s): David Jordan (dj333@cam.ac.uk)

Review Timeline:

Submission Date:	21st Jun 23
Editorial Decision:	10th Aug 23
Revision Received:	29th Aug 23
Editorial Decision:	14th Sep 23
Revision Received:	26th Sep 23
Accepted:	5th Oct 23

Editor: Maria Polychronidou

Transaction Report:

(Note: With the exception of the correction of typographical or spelling errors that could be a source of ambiguity, letters and reports are not edited. Depending on transfer agreements, referee reports obtained elsewhere may or may not be included in this compilation. Referee reports are anonymous unless the Referee chooses to sign their reports.)

10th Aug 2023

Manuscript Number: MSB-2023-11835
Title: Canalisation and plasticity on the developmental manifold of Caenorhabditis elegans

Dear Dr. Jordan,

Thank you again for submitting your work to Molecular Systems Biology. We have now heard back from the three reviewers who agreed to evaluate your study. As you will see below the reviewers acknowledge that the study seems relevant for the field. They do however raise a series of concerns which we would ask you to address in a revision.

I think that the reviewers' comments seem rather clear and I therefore do not see the need to repeat any of them here. As reviewer #2 indicates, it would be important to revise the manuscript for clarity and to ensure that it is easily accessible to a broad audience. All issues raised by the reviewers need to be satisfactorily addressed. As you may already know, our editorial policy allows in principle a single round of major revision, so it is essential to provide responses to the reviewers' comments that are as complete as possible. Please feel free to contact me in case you would like to discuss in further detail any of the issues raised. I would be happy to schedule a call.

On a more editorial level, we would ask you to address the following points:

- Related to the points of reviewers #1 and #3 on the Boxes: we do allow Boxes and we would recommend keeping them both in. They can be especially useful for a broader audience.

- We typically do not have a separate "Conclusions" section, so we would ask you to incorporate it in the Discussion.

- Please provide a .doc version of the manuscript text (including legends for the main figures and EV Figures) and individual production quality figure files for the main Figures and EV Figures (one file per figure).

- We have replaced Supplementary Information by the Expanded View (EV format). In this case, all additional figures* can be provided as EV Figures. Please provide one file per EV Figure. Their legends should be included in the manuscript text.
*If after the performed revisions the number of additional Figures is > 6, then they can all be included in a PDF called Appendix. Appendix figures should be labeled and called out as: "Appendix Figure S1, Appendix Figure S2... Appendix Table S1..." etc. Each legend should be below the corresponding Figure/Table in the Appendix. Please include a Table of Contents in the beginning of the Appendix. For detailed instructions regarding expanded view please refer to our Author Guidelines: .

- Please include 5 keywords.

- Please provide a "standfirst text" summarizing the study in one or two sentences (approximately 250 characters), three to four "bullet points" highlighting the main findings and a "synopsis image" (550px width and max 400px height, jpeg format) to highlight the paper on our homepage.

- All Materials and Methods need to be described in the main text. We would encourage you to use 'Structured Methods', our new Materials and Methods format. According to this format, the Materials and Methods section should include a Reagents and Tools Table (listing key reagents, experimental models, software and relevant equipment and including their sources and relevant identifiers) followed by a Methods and Protocols section in which we encourage the authors to describe their methods using a step-by-step protocol format with bullet points, to facilitate the adoption of the methodologies across labs. More information on how to adhere to this format as well as downloadable templates (.doc or .xls) for the Reagents and Tools Table can be found in our author guidelines: . An example of a Method paper with Structured Methods can be found here:

- Please include a "Disclosure and Competing Interests Statement" in the main text.

- Please include a Data availability section describing how the data, code etc. have been made available. This section needs to be formatted according to the example below:
The datasets and computer code produced in this study are available in the following databases:
- Chip-Seq data: Gene Expression Omnibus GSE46748 (https://www.ncbi.nlm.nih.gov/geo/query/acc.cgi?acc=GSE46748)
- Modeling computer scripts: GitHub (https://github.com/SysBioChalmers/GECKO/releases/tag/v1.0)
- [data type]: [full name of the resource] [accession number/identifier] ([doi or URL or identifiers.org/DATABASE:ACCESSION])

- For data quantification: please specify the name of the statistical test used to generate error bars and P values, the number (n) of independent experiments (specify technical or biological replicates) underlying each data point and the test used to calculate p-values in each figure legend. The figure legends should contain a basic description of n, P and the test applied. Graphs must

include a description of the bars and the error bars (s.d., s.e.m.).

- The References should be formatted according to the Molecular Systems Biology reference style (i.e. ordered alphabetically and listing the first 10 authors followed by et al).

- When you resubmit your manuscript, please download our CHECKLIST (https://bit.ly/EMBOPressAuthorChecklist) and include the completed form in your submission.
Please note that the Author Checklist will be published alongside the paper as part of the transparent process (https://www.embopress.org/page/journal/17444292/authorguide#transparentprocess).

If you feel you can satisfactorily deal with these points and those listed by the referees, you may wish to submit a revised version of your manuscript. Please attach a covering letter giving details of the way in which you have handled each of the points raised by the referees. A revised manuscript will be once again subject to review and you probably understand that we can give you no guarantee at this stage that the eventual outcome will be favorable.

Kind regards,

Maria

Maria Polychronidou, PhD
Senior Editor
Molecular Systems Biology

We realize that it is difficult to revise to a specific deadline. In the interest of protecting the conceptual advance provided by the work, we recommend a revision within 3 months (8th Nov 2023). Please discuss the revision progress ahead of this time with the editor if you require more time to complete the revisions. Use the link below to submit your revision:

https://msb.msubmit.net/cgi-bin/main.plex

IMPORTANT: When you send your revision, we will require the following items:
1. the manuscript text in LaTeX, RTF or MS Word format
2. a letter with a detailed description of the changes made in response to the referees. Please specify clearly the exact places in the text (pages and paragraphs) where each change has been made in response to each specific comment given
3. three to four 'bullet points' highlighting the main findings of your study
4. a short 'blurb' text summarizing in two sentences the study (max. 250 characters)
5. a 'thumbnail image' (550px width and max 400px height, Illustrator, PowerPoint or jpeg format), which can be used as 'visual title' for the synopsis section of your paper.
6. Please include an author contributions statement after the Acknowledgements section (see https://www.embopress.org/page/journal/17444292/authorguide)
7. Please complete the CHECKLIST available at (https://bit.ly/EMBOPressAuthorChecklist).
Please note that the Author Checklist will be published alongside the paper as part of the transparent process (https://www.embopress.org/page/journal/17444292/authorguide#transparentprocess).
8. When assembling figures, please refer to our figure preparation guideline in order to ensure proper formatting and readability in print as well as on screen:
https://bit.ly/EMBOPressFigurePreparationGuideline
See also figure legend guidelines: https://www.embopress.org/page/journal/17444292/authorguide#figureformat
9. Please note that corresponding authors are required to supply an ORCID ID for their name upon submission of a revised manuscript (EMBO Press signed a joint statement to encourage ORCID adoption).
(https://www.embopress.org/page/journal/17444292/authorguide#editorialprocess)
Currently, our records indicate that the ORCID for your account is 0000-0002-3506-5440.

Please click the link below to modify this ORCID:
Link Not Available

10. At EMBO Press we ask authors to provide source data for the main manuscript figures. Our source data coordinator will contact you to discuss which figure panels we would need source data for and will also provide you with helpful tips on how to upload and organize the files.

The system will prompt you to fill in your funding and payment information. This will allow Wiley to send you a quote for the article processing charge (APC) in case of acceptance. This quote takes into account any reduction or fee waivers that you may be eligible for. Authors do not need to pay any fees before their manuscript is accepted and transferred to the publisher.

EMBO Press participates in many Publish and Read agreements that allow authors to publish Open Access with reduced/no publication charges. Check your eligibility: https://authorservices.wiley.com/author-resources/Journal-Authors/open-access/affiliation-policies-payments/index.html

As a matter of course, please make sure that you have correctly followed the instructions for authors as given on the submission website.

*** PLEASE NOTE *** As part of the EMBO Press transparent editorial process initiative (see our Editorial at https://dx.doi.org/10.1038/msb.2010.72), Molecular Systems Biology publishes online a Review Process File with each accepted manuscripts. This file will be published in conjunction with your paper and will include the anonymous referee reports, your point-by-point response and all pertinent correspondence relating to the manuscript. If you do NOT want this File to be published, please inform the editorial office at msb@embo.org within 14 days upon receipt of the present letter.

Reviewer #1:

"Canalisation and plasticity on the developmental manifold of Caenorhabditis elegans" by Jordan and Miska uses quantitative data on C. elegans development collected from different strains fed different foods to look at the nature of phenotypic variation. Specifically they look at growth rates, timings of several developmental events, and final size. They then consider correlations between these parameters and show that there within-population and between-population correlations are related. For example, they confirm a recently reported negative correlation between growth rate and the duration of development. Jordan and Miska show that similar relationships hold for other phenotype pairs as well. Finally, they show that further dimensionality reduction using nonlinear PCA reveals a 2D manifold whose dimensions partition the variance into environmental and genetic components.

This work is important as it sits in the context of longstanding work on phenotypic spaces, genotype-phenotype maps, and how the geometry of these spaces relates to evolution and quantitative genetics dating back to Fisher. This paper presents fresh data and a fresh approach. One of the strengths of the paper is that it combines experiments and analysis with a conceptual, even pedagogical, approach to the problem, making it a pleasure to read. In some ways it poses more questions than it answers, but I don't consider this a weakness. This is the kind of paper that can help start a new subfield by spurring further experimental and theoretical work. Again, the clarity and presentation are outstanding and improve the odds of others taking up related projects.

I don't know if MSB normally allows boxes. I hope they can stay in, the format really works here.

In Figure 4A-D it would be nice to see where the mutant worms sit. As the authors say when discussing previous work, the results could depend on how "populations included in the analysis sample natural genetic variation, genetic mutations, environmental variations, or combinations of all three." Because they have already sampled some natural and artificial genetic variation, I would be curious to see if the mutants violate the trend seen for wild isolates. If you keep the manifold as is, but add the mutant data to Figure 4A, do they sit off the manifold? If not, do they violate the trends for genetic and environmental variation seen in D and E?

In Figure 3E, since there aren't very many points, would it be possible to annotate them with the trait pairs the represent? If that's too busy, perhaps just annotating the extremes of positive and negative correlation would still be useful? Another alternative would be to include a larger annotated version in the supplement.

Despite the excellent writing over all, there are some minor typos and missing words that should be fixed. An incomplete list:

-"between the directions of fluctuations in a fixed context the directions in which populations will shift" Missing 'and' between context and the?

-"grown on differ food sources" differ -> different

-"variation in individuals to be correated in the same way as variations between populations". Should this be variation between individuals? Or phrased as variation within populations vs between populations?

-I would spell out the single use of NLPCA.

-"non-linear principle com- ponents" principle -> principal

-"We propose that is natural to view" is natural -> it is natural

-"comprised of of ≈ 3700 genes" remove one 'of'

-"the incredibly diversity" incredibly -> incredible

Reviewer #2:

Manuscript draft title: Canalisation and plasticity on the developmental manifold of Caenorhabditis elegans

In this ms draft the authors describe a method for studying the structure of variability in the development of an animal model. The main mode of analysis is by correlation, but it is not clear what type of correlation they use. They state that the geometric structure of these correlations can be captured by a low dimensional manifold. The authors state that their framework provides a basis for both robustness and plasticity.

Major points:

This ms was hard to follow. That is, while there is a new way to quantitatively analyze phenotypic space that is explored in ways to test variance within and between C. elegans populations, what is not clear is the sensitivity and specificity of the approach.

The manuscript speaks in a general and vague style making it hard for the reader to understand the main message and final result. It is unclear how the main results link up with the claims in the title and abstract.

This ms tries to express concepts in analysis of canalization and plasticity, yet there are terms used here that look like jargon not fully described: eg the word manifold appears 27 times but apart from a sense of it as dimensionality reduction term it is not clear how it is so central to this particular set of questions. Other terms are even more confusing, eg "concentration of dimension" (see abstract, introduction - 3 times, and discussion).

The authors use the following explanation in the introduction, but it is still not clear what their message is here.
Concentration of dimension may be an intrinsic property of systems where robustness and control of the macroscopic observables are required [17] and may arise from constraints imposed by steady state growth [18, 19], or trade-offs between phenotypic archetypes suited for different tasks [20].

Could the authors elaborate on the meaning of "projection" in the context of this work?

They also use the word "context" at least 13 times but do not elaborate on the context itself. This leaves the reader wondering what the author is referring to? For example - Box 2: "even in very divergent contexts" what does this mean to the author?

Another example in Box2: "...the prior distribution over contexts,..." what does this mean?

The abstract states the following - but what does it mean?
Our perspective offers a new way of understanding the relationship between robustness and flexibility in complex systems, suggesting that projection and concentration of dimension can naturally align these forces as complementary rather than competing.

The discussion is so broad and non-specific making it difficult to understand what message they are trying to convey. For example - "At the heart of concentration of dimensionality lies projection [what does this mean?]. We propose that is natural to view robustness and plasticity as analogous to projection of variation onto, or orthogonal to, a low dimensional manifold [what does this mean?].

Canalisation and developmental plasticity seem to be opposing forces. How so? Can they elaborate and add context? Is this what was discovered in their analysis?

In the conclusion the authors repeatedly say "context" - "they observed the growth of the nematode in environmental and genetic contexts to map the variability of development. We find that traits which are correlated within a particular context predict whether the mean values of those traits will be correlated among different contexts." - Can the authors be more specific? Use examples?

What is the control group in this experiment? Is there a control group for both - within population analysis and between population analysis?

Fig 2 (b) is the normalized data, but after normalization all conditions look the same (is this expected?)

What data is being read into the NLPCA? How many dimensions of data? Number of rows/samples? Number of columns/features? Are the data numerical? Continuous or discrete?

Why specifically did they use NLPCA? Did they compare their results to other methods? Regular PCA? UMAP? Tsne?

An advantage of nonlinear PCA over linear PCA is the ability to incorporate nominal and ordinal variables, and that it can handle and discover nonlinear relationships between these variables. Are the authors using any categorical data here?

Page 8 Results/Fig 4a - The authors state that NLPCA was used to determine the shape of this ambient space of rescaled logistic parameters with the developmental duration included. It is unclear to me what data was used in the NLPCA analysis - how can you feed in logistic parameters to perform any sort of PCA?
Could the authors also explain what an "ambient phenotypic space" is?

Figure 4(a) - Could the authors elaborate more on figure 4a? This seems to be the major result of this paper, yet they dedicate only 1 - 2 sentences toward this result. What do the x,y,z, axis represent? Shouldn't they represent the principal component dimensions? Are these the first three extracted non-linear components?

FIgure 4(a) Could the authors elaborate on each principal component, by providing some sort of interpretation in the context of the data being used?
Figure 4(a) the figure caption says 2-D manifold, but the plot is 3-D.

What are the curves in Fig 4b? What does the y - axis range represent? Are these the latent space projected onto 2D space?

Fig 4 (e) legend says "mixed mixed" regression - what is "mixed mixed" regression?

Page 8 Fig 4 - "Within the embedding of growth curves, animals tend to cluster in phi_1 according to their food source" Is 4(b) meant to illustrate clusters? What is the meaning of a cluster in this context? Clusters usually means groups of individuals behaving in the same way.

Figure 4: What is the meaning of phi_1 and phi_2 ?

Conclusion states that "Correlation between traits indicate that the true dimensionality of the system may be lower" but how is such a claim supported in this manuscript?

They authors claim that genetic variation on the phenotype are separable from those of environmental variation. How is this true?

Regarding the outcome of this study:

What was the actual outcome of this study? Did the authors identify the dominant mode of phenotypic variation?

The authors don't compare to other methods, nor elaborate on how this is more widely applicable

Questions about methods/data/experimental conditions

Can the authors show the data? The measured variables/prediction variables? Is this available?
Is the matlab code available for the NLPCA analysis?
Is the raw data and normalized data available?

Can the authors provide a table which summarizes the variables/measurements being used in this study? Which variables are being measured manually or automatically, which variables are derived from logistic fit?

In the results it states that length and area are measured automatically, but egg hatching and first laying egg are recorded manually? Which variables are recorded manually versus automatically?
How many variables are being measured and recorded? Two?
Is reproductive maturation another variable? If so, what is this a measure of?
Are the perturbations/conditions "natural genetic variation" and "single gene mutant"? Are these the two main environmental conditions?
Is "wild isolates"/"natural selection another condition?
Diet conditions: different kinds of bacteria

Minor points:

In the introduction (line numbers are not present) it says ..."and manually recording" - should this be "recorded"?
Fig 1 - are there 8 or 10 time points in (a) ? I only see 8 time points.
Fig 1(c) says 10 specific points highlighted, but I only see 8 points.
Page 5 says "differ food sources differ..." Should it say "different"?
Box 2: do the authors cite the method (discrete cosine transformation) they use?
Are the authors making a connection between Box 2 and canalisation/plasticity of c elegans?
Fig S2: (b) shouldn't the means (+) be different colors? Shouldn't the colors correspond to the colors in (a).

Fig S3: (a) how many individuals? (b) What are we supposed to be able to see in the heatmap/spatial distributions?

Is there a table explaining all the different conditions? (see legend showing conditions) in Fig S3 (a).

What do the different colors represent in Figure S2 (a)?
The authors use phrases such as "cryptic variation" (see Discussion), what does this mean?
"Incredibly diversity" in the Conclusion must be a typo?

Reviewer #3:

Report of the paper "Canalisation and plasticity..." by Jordan and Miska
In the paper the authors study the homeorhesis of development of C elegans. By studying the growth curve on various conditions and mutants, they unveiled low-dimensional phenotypic manifold. The results are interesting and the experimental analysis is clearly presented. I recommend the publication of the manuscript after the authors consider the following points. Some comments are a bit idiosyncratic, so these are for optional for revision of the present manuscript, but maybe for future studies.
1) The term homeorhesis was originally coined by Conrad Waddington, together with his renown landscape, so it will be good to mention this explicitly. Also, there are some theoretical studies for homeorhesis in developmental process (e.g., ref [1]).
2) In contrast to the parameters l^{hat} and r^{hat} thoroughly investigated, the parameter A was less discussed. It will be good to discuss a bit more the meaning of A. At around t\sim A, the growth speed is maximal. Is there some biological meaning for it? Compared with l^{hat} or r^{hat}, how about is the degree of robustness (or variance) in A?
3) Does the magnitude of variance of l just simply increase with development time? Or is there some bottleneck time as discussed in the hourglass picture? (This may depend on the way to select egg, though. --I wonder if the initial variance in natural condition may be larger, and can be buffered in the middle stage in development).
4)In the discussion, the authors mention that the origin of low-dimensional dynamics and of the fluctuation-response relationship remains a mystery. I agree that it is not solved. Still, there is some discussion on it, albeit it may be hand-waiving. In Furusawa and Kaneko ([2],) it is discussed, by assuming the robustness of the developed phenotype to perturbations, strong attraction to the state follows, while plasticity is kept in the direction of evolution, as the phenotype should change through the evolution. Then, the variances by noise (or environment) and by mutation follow the common manifold, leading to the correlation (proportionality)between the two [3], from which evolutionary fluctuation relationship follows.
Now, in the present study, from the nonlinear PCA, the direction for plastic change is extracted, so that one may consider in which direction evolutionary plasticity is kept. Such discussion may be of biological significance.
5) Also, in the above sense, it will be interesting to explore possible relationship between the phenotypic variance by noise and by mutation (even though the phenotypes measured here are just three; l^{hat}, r^{hat} and A).
6) I am not completely sure if the Box 2 is necessary. Still probably it will be useful to most biology readership, so I will leave the judgment to the editor.
7) The discussion on possible relationship in gene expression oscillator is interesting. According to the experiment the period is about 8 hours. It is smaller than A\sim 30hours, but in their experiment oscillation ceases by SNIC bifurcation after about a 4-5 periods or so. It may be useful explore this point, if possible. (In fact in ref [1], indeed, oscillatory dynamics provide homeorhesis).
Sincerely yours, Kunihiko Kaneko
[1]Matsushita et al., Phys. Rev. Research 2, 023083(2020))
[2]Furusawa and Kaneko, Physical Review E 97.4 (2018): 042410.
[3]Furusawa and Kaneko, Journal of The Royal Society Interface 12.109 (2015): 20150482.

NOTES TO THE EDITOR

Dear Maria,

Thanks very much for the work you have done as editor of our manuscript, it has been a pleasure working with you and with Molecular Systems Biology and we are very happy to support the EMBO. The review process has been a wholly positive experience and I think the manuscript is much better for it. You will find below a list of points that we have addressed in the revisions that are of primarily editorial concern.

1. The entire document has been reformatted as a word document, and references have been redone in the EMBO press style. References include a DOI separated from each entry by a space.
2. In addition to the typos pointed out by the reviewers, I have carefully read the manuscript to remove any remaining errors.
3. I have merged the Conclusions into the Discussion section.
4. In response to reviewer comments (Reviewer 1 Comment 2, Reviewer 3 Comment 5) I performed some additional analysis, which turned out to be very interesting. As such, I have generated a new figure (Fig 5) to include this analysis in the MS, as well as a paragraph in the results describing it. I hope this is ok. I can make it an EV figure, but I thought that because these are not included inline in the PDF version of the article, these additional results warranted a main figure.
5. I have included a .doc file of the main text and separate .eps files for each Figure and EV Figure.
6. I have prepared source data files for each Figure. These include CSV files of the numerical data required to generate each plot. To further aid replication, I have uploaded all functions and scripts required to go from raw data to figure on GitHub as mentioned in the text. In addition, each source data file contains a script that shows how the CSV files were generated and takes the CSV file data and plots it as it is plotted in each figure panel (requires MATLAB). (@ Hannah Sonntag)
7. The methods have been reformatted as Structured Methods with a Reagents and Tools table and a Methods and Protocols text section.
8. I have included the keywords in the main text file, as well as the standfirst text and synopsis image.
9. I have included a disclosures and competing interests statement and a data availability statement in the main text.
10. I have completed and uploaded the author checklist.

GENERAL AUTHOR COMMENTS TO REVIEWERS

I would like to thank all of the reviewers for their timely and useful comments. A paper of this sort is difficult to write and can easily be the worst of both worlds for theorists and experimentalists reading it. I hope I have avoided that as much as possible. I was very encouraged by your comments. Furthermore, the additional analysis that I have done has resulted in both a new figure for the manuscript and some nice explanatory examples. The comments have seemed to me to be in very good faith, and the whole review process has, in my opinion, improved the manuscript greatly. Kind regards, David

REVISIONS IN REFERENCE TO REVIEWER CONCERNS WITH AUTHOR RESPONSES

NUMBERED LIST OF REVIEWER COMMENTS:

Reviewer 1

"Canalisation and plasticity on the developmental manifold of Caenorhabditis elegans" by Jordan and Miska uses quantitative data on C. elegans development collected from different strains fed different foods to look at the nature of phenotypic variation. Specifically, they look at growth rates, timings of several developmental events, and final size. They then consider correlations between these parameters and show that there within-population and between-population correlations are related. For example, they confirm a recently reported negative correlation between growth rate and the duration of development. Jordan and Miska show that similar relationships hold for other phenotype pairs as well. Finally, they show that further dimensionality reduction using nonlinear PCA reveals a 2D manifold whose dimensions partition the variance into environmental and genetic components.

This work is important as it sits in the context of longstanding work on phenotypic spaces, genotype-phenotype maps, and how the geometry of these spaces relates to evolution and quantitative genetics dating back to Fisher. This paper presents fresh data and a fresh approach. One of the strengths of the paper is that it combines experiments and analysis with a conceptual, even pedagogical, approach to the problem, making it a pleasure to

read. In some ways it poses more questions than it answers, but I don't consider this a weakness. This is the kind of paper that can help start a new subfield by spurring further experimental and theoretical work. Again, the clarity and presentation are outstanding and improve the odds of others taking up related projects.

1. I don't know if MSB normally allows boxes. I hope they can stay in, the format really works here.
   Thanks, they are allowed, and I have kept them in.
2. In Figure 4A-D it would be nice to see where the mutant worms sit. As the authors say when discussing previous work, the results could depend on how "populations included in the analysis sample natural genetic variation, genetic mutations, environmental variations, or combinations of all three." Because they have already sampled some natural and artificial genetic variation, I would be curious to see if the mutants violate the trend seen for wild isolates. If you keep the manifold as is, but add the mutant data to Figure 4A, do they sit off the manifold? If not, do they violate the trends for genetic and environmental variation seen in D and E?
   Thanks for this comment! I had originally neglected an in-depth analysis of the mutants, as I had not tested them on all the different bacterial diets, only the lab strain of *E. coli*. However, in preparing my reply to the reviewer's questions, I have found some very interesting results regarding the mutants and have included this analysis as a new Figure 5. The two mutants that I included were a deletion of the sid-2 gene, which is an otherwise healthy animal and whose main phenotype is the loss of the ability to do RNAi by feeding. We had previously seen that this mutation slightly accelerated development and led to slightly longer animals (but not as long as long mutants such as *lon-2*). The other mutant is a deletion in a putative helicase that had a severe developmental delay with worms much smaller and "sicker". Interestingly, this severe mutant does indeed break trends in diet and genotype. Although the mild mutant overlaps significantly with the distributions for other N2 worms and for worms fed *E. coli*, the C28 mutant is very far from other N2 worms and from other worms fed *E. coli*. In addition, if we look at how "off manifold" the mutants are, by measuring their average position along the third nonlinear principal component ϕ_3 in fact we do find that both mutants are off the manifold (positive in the ϕ_3 direction). It is interesting to note that the *sid-2* mutant is off-manifold in the same way as the C28 mutant despite it having a mild phenotype. The wild-isolate strains AB1 and PS2025 are also slightly off-manifold, but in the opposite direction (negative ϕ_3) to the mutants! Furthermore, while the mutants are off-manifold fed on E. coli, the wild-isolates are only off-manifold when their diets are perturbed as well. In addition to the new Figure 5 and the caption of the figure, I have included a paragraph in the results that describes these findings, as I have related them to you above.
3. In Figure 3E, since there aren't very many points, would it be possible to annotate them with the trait pairs the represent? If that's too busy, perhaps just annotating the extremes of positive and negative correlation would still be useful? Another alternative would be to include a larger annotated version in the supplement.
   The annotations were indeed a bit busy on the main plot, but I have included them in an extended view (supplemental) plot. This has the added benefit that I can include some more trait pairs in the correlation plot and thus I have included 2 more traits, l_{hatch} the length of the newly hatched worm and l_{dev} the length of the adult worm when it lays its first egg. This results in 7 traits and 21 unique trait pairs.
4. Despite the excellent writing overall, there are some minor typos and missing words that should be fixed. An incomplete list:
   a. -"between the directions of fluctuations in a fixed context the directions in which populations will shift" Missing 'and' between context and the?

   Line 120 Added the following text: *and*

   b. -"grown on differ food sources" differ -> different

   Line 194 Changed the following text: *differ* to *different*

   c. -"variation in individuals to be correlated in the same way as variations between populations". Should this be variation between individuals? Or phrased as variation within populations vs between populations?

   Line 201 Change *in individuals* to *within populations*

   d. -I would spell out the single use of NLPCA.

   Line 225 Changed the following text: *NLPCA parameters* to *nonlinear principal component embedding*

   e. -"non-linear principle com- ponents" principle -> principal

Line 239 Changed the following text: *principle* to *principal*

f. -"We propose that is natural to view" is natural -> it is natural

Line 291 Added text: *it*

g. -"comprised of of ≈ 3700 genes" remove one 'of'

Line 367 Removed text: *of*

h. -"the incredibly diversity" incredibly -> incredible

Line 392 Changed text: *incredibly* to *incredible*

Reviewer 2

In this ms draft the authors describe a method for studying the structure of variability in the development of an animal model. The main mode of analysis is by correlation, but it is not clear what type of correlation they use. They state that the geometric structure of these correlations can be captured by a low dimensional manifold. The authors state that their framework provides a basis for both robustness and plasticity.

1. This ms was hard to follow. That is, while there is a new way to quantitatively analyze phenotypic space that is explored in ways to test variance within and between C. elegans populations, what is not clear is the sensitivity and specificity of the approach.

   It is not clear where the sensitivity and specificity analysis would apply to this work. I am familiar with these terms in the context of classification problems, and with their relation to methods such as receiver operator characteristic (ROC) curves and associated quantification such as Area Under ROC (AUROC), but because we are not doing any classification, I am unsure for what purpose we would calculate true positive rates and false positive rates here. For the sake of this response, I have devised a hypothetical classification problem we could address with these data, but as it is not one of the focusses of our manuscript, I will not add it to the MS and leave it only for this letter.

   Let us assume that we would like to classify whether an animal was fed *Bacillus* or *Pseudomonas*. Classification is mathematically related to clustering, so it may help to refer to my answer to Question 20 below. For this hypothetical situation, let us attempt to classify these two groups based on their ϕ_1 Value and compare this to classification using each of the three logistic fit parameters.

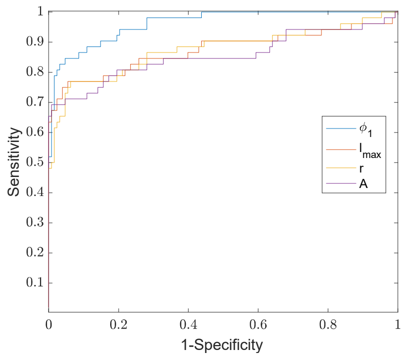

   Because these two conditions cluster separately in ϕ_1 we would expect this to be a useful parameter for classification. As we increase the threshold on ϕ_1, assigning individuals below the threshold to *Pseudomonas* and those above to *Bacillus*, we see that more of the *Pseudomonas* fed individuals are correctly identified (sensitivity goes up), however, we don't start misclassifying *Bacillus* fed individuals until we reach a threshold value of around 0, where we have 80% sensitivity. We can choose some sort of optimal threshold if we have a sensitivity we wish to achieve or a specificity we are unwilling to go below. In this case I have chosen 0.0561, where we have 90% sensitivity and 85% specificity. Compared to using the raw logistic fit parameters for classification, at a similar sensitivity, the specificity is much lower, at 56%, 55%, and 36% for I_max, r, and A, respectively. This sort of analysis is also captured in the F-statistic presented in Fig4E, but instead of looking at each individual classification problem for each pair of groups, the F-statistic summarises the classifiability for all the pairs of groups together. We can tell quantitatively that ϕ_1, is a better parameter for classifying these two foods with the Area Under the ROC, which for these four classifiers is [0.94 0.85 0.85 0.83].

2. The manuscript speaks in a general and vague style making it hard for the reader to understand the main message and final result. It is unclear how the main results link up with the claims in the title and abstract.
   I apologize for the general and vague style. I had originally intended to include a full theoretical treatment of projection operators, fluctuation dissipation relations, and Hilbert spaces of functions in this manuscript so that I could discuss these things precisely instead of generally, but on advice from even specialized readers it made the manuscript to onerous to read for both biology and physics audiences. Therefore, I decided to remove the theoretical discussion for another companion manuscript. The result however, is that I have to speak more vaguely about these topics than I would like. I have included some points from the discussion, particularly about projection operators, in the responses to your questions below. While I still feel it would not work well to include it in the main MS at this time, I hope that it is sufficient to make the discussion more concrete for you. I am have compiled these points as well into the Appendix of this paper and plan to elaborate on them in an accompanying blog post or similar less formal discussion medium to be made publicly available.
3. This ms tries to express concepts in analysis of canalization and plasticity, yet there are terms used here that look like jargon not fully described: eg the word manifold appears 27 times but apart from a sense of it as dimensionality reduction term it is not clear how it is so central to this particular set of questions. Other terms are even more confusing, eg "concentration of dimension" (see abstract, introduction - 3 times, and discussion).
   I have included a new paragraph in the introduction (Line 48) that describes in more detail the terms that may not be familiar to a more general audience. The use of jargon is always a trade-off; it is harder for a broad audience to understand, but it greatly increases the efficiency of communication for specialists. I have tried to use it where appropriate to strike a balance between these. It can be extremely beneficial to express ideas succinctly that are very difficult to explain, such as your use of Latent Space in Question 18. Anyone familiar with this machine learning terminology will instantly have a rich understanding of what you mean by latent space; I immediately know what you are referring to when you use it, which makes our discussion more efficient. In particular, I have introduced "concentration of dimension" as a new piece of jargon precisely for this reason. I wanted to have a term that conveys the ideas of dimensionality reduction, but not be construed with *techniques* for finding low-dimensional representations like PCA, t-SNE, UMAP, etc. For example, when talking about a bottleneck autoencoder, we can now talk about the concentration of dimension that happens in the encoding stage to the latent space. This is not precisely a dimensionality reduction technique *per se* (although it can be used for dimensionality reduction and is how the NLPCA is done in this work), it is more of a property of the network in how different layers are wired together. I would like to evoke the idea of the emergence of low-dimensional modes from the interactions of many components in higher dimensions. One nice example that is physically motivated is the concentration of dimension that emerges from the elastic network of amino acid interactions in a protein.
4. The authors use the following explanation in the introduction, but it is still not clear what their message is here. Concentration of dimension may be an intrinsic property of systems where robustness and control of the macroscopic observables are required [17] and may arise from constraints imposed by steady state growth [18, 19], or trade-offs between phenotypic archetypes suited for different tasks [20].
   Here we note that concentration of dimension is found throughout biological systems and point to some recent work that speculates why it is found and how it is generated. The main message is that other have observed this phenomenon and have proposed explanations for its existence.
5. Could the authors elaborate on the meaning of "projection" in the context of this work?
   Of course! In the context of this work, projection refers to a general class of idempotent functions (maps) from a set (space) into a subset (subspace). Idempotent means that repeated applications after the first do not further alter the result. If P is a projection map, this means that $P\big(P(x)\big) = P(x)$ In particular we are interested in projections in Euclidean spaces (linear projections and projections onto manifolds) and also projections in Hilbert spaces. The most familiar projection operator may be the projection onto the canonical basis in the n-dimensional Euclidean space $\mathbb{R}^n$, which is a linear operator. If we have a vector $\vec{v}$

in $\mathbb{R}^n$, the projection map is the inner (dot) product with the canonical basis. For example the projection onto the “x” axis is given by:

$$P_{e_1}(\vec{v}) = \vec{v} \cdot e_1 = \vec{v} \cdot [1,0,0,0 \ldots] = v_1$$

In a Hilbert space of functions, we have an analogous projection function. This is easiest to demonstrate if we have an orthonormal set of basis functions, for example, the periodic basis of the Fourier transform. In this case, the projection is still given by the inner product, but the inner product has a different form,

$$\mathrm{f} \cdot \mathrm{g} = \langle \mathrm{f}, \mathrm{g} \rangle = \frac{1}{2\pi} \int_0^{2\pi} \mathrm{f}(\mathrm{x}) \mathrm{g}^*(\mathrm{x}) \mathrm{dx}$$

We can think of any function as a vector in this space. If we call the set of basis functions, ϕ_i then any function p can be represented as a vector of weights $p = \sum_{i=1}^{\infty}(w_i \phi_i)$ with the weights given by $w_i = \langle p, \phi_i \rangle$. We can then further ‘project’ this function onto a subset of basis functions using the standard dot product with the weight vector w_i. In practice, because we are using an orthonormal basis, this is equivalent to truncating the infinite sum. This projection is often required in real world problems as the weight vector is in principle infinite dimensional and must be approximated by a finite number of basis functions. Projection operators on infinite dimensional Hilbert spaces may seem removed from biological problems, but they arise in statistical mechanics in physical problems that are similar in some respects to biological problems. An example is the diffusion of a drop dye in water. In principle, the dynamics that govern the time evolution of the concentration of the dye as function of depends on all the position and momenta of the 10^{23}particles involved. However, the dynamics of the concentration can be described well as a function of the concentration itself. This derivation can be made exactly using projection operators (See e.g. Zwanzig R *Nonequilbirum Statistical Mechanics* Oxford University Press, 2001). I had originally wanted to include a review of this theory in this paper, but as you’ve pointed out it is already bard to follow so I decided to separate it out into a separate theory paper. I will be happy to send you a draft of this MS when it is prepared if you are interested. I have included this discussion in the Appendix as well.

6. The discussion is so broad and non-specific making it difficult to understand what message they are trying to convey. For example –
    a. "At the heart of concentration of dimensionality lies projection [what does this mean?].
    This is a general mathematical statement about projection maps. Projection functions map a space into a subspace.
    b. We propose that is natural to view robustness and plasticity as analogous to projection of variation onto, or orthogonal to, a low dimensional manifold [what does this mean?].
    To answer this question, I will refer to my answer to Question 14. It may be helpful to read that first.

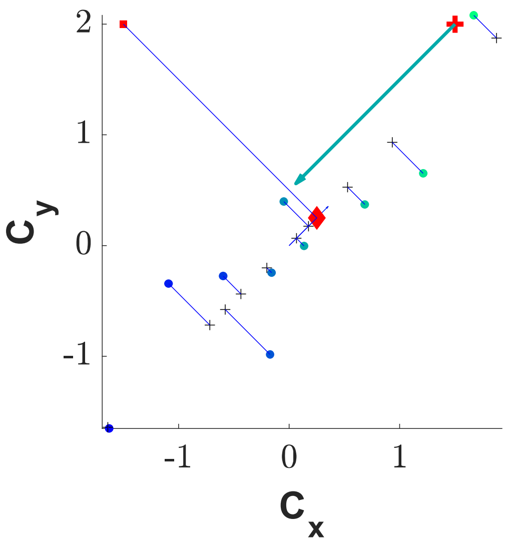

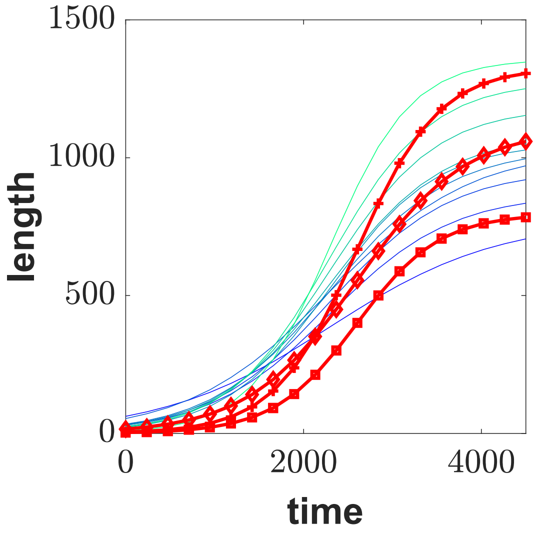

Imagine we have a molecular process that generates values for Cx and Cy and which converts those signals into a developmental trajectory, as in Answer 14. Suppose a mutation generates an aberrant expression pattern which results in the red square above. In the absence of projection, or of concentration of dimension as we have called it, this aberrant expression pattern would result in a very different developmental trajectory (red line with squares in the right panel). However, under the action of the projection function, this aberrant expression point is mapped to its location on the manifold (in this case the line Cy=Cx) which gives the reduced dimensional representation indicated by the red diamond in the left figure. The action of this projection operator results in a growth curve (right panel, red diamond line) this is much less aberrant. Thus, projection onto the manifold has endowed the system with robustness. However, the projection has not completely buffered the developmental trajectory from this mutation. If we look at the difference between the *wild-type* developmental trajectory (red plus line right panel), and the projected mutant developmental trajectory (red diamond line, right panel), there is still variation that corresponds the component of the projection *along* the manifold, as shown by the teal arrow pointing from the red plus to the red diamond in the left panel. Thus, projection parallel to the manifold allows for plasticity, but the nature of this plasticity is constrained. The removal of the component orthogonal to the manifold constrains it and this results in robustness. I have included this discussion in the Appendix.

7. Canalisation and developmental plasticity seem to be opposing forces.
   a. How so?
   Canalization is a process which reduces of phenotypic or developmental variation, while plasticity increases variability. By definition they appear to be opposing forces.
   b. Can they elaborate and add context?
   The fact that by definition they are opposing forces, yet biology seems to be both robust and plastic was one of the paradoxes that led us to this study. If I wished to build a physical system that was very robust, I would strive to make its function independent of external conditions, of its environment, and of any stochastic failures of its parts. How would such a system also be able to respond to the environment, diversify the functions it carries out, and adapt to changing conditions? This seems to be a difficult engineering problem, yet one that biology has solved.
   c. Is this what was discovered in their analysis?
   What we have discovered, or proposed rather, is a design principle which may lend both robustness and flexibility to a complex system.
8. In the conclusion the authors repeatedly say "context" - "they observed the growth of the nematode in environmental and genetic contexts to map the variability of development. We find that traits which are correlated within a particular context predict whether the mean values of those traits will be correlated among different contexts." - Can the authors be more specific? Use examples?
For example, if we only measured l_{max} and r in the context of N2 animals fed *E. coli*, we would find a strong negative correlation between these traits. We might then predict if we take some animals of an unknown genotype and fed an unknown diet and find that they are much longer at adulthood, even without measuring their full development, we might propose that their maximal growth rate is less. In addition, because we have found that this is indeed true for these traits among the combinations of food and genotype we have assayed in this study, we might propose that there is some underlying mechanism that results in this negative correlation, similar to the one proposed in Box 1.
9. What is the control group in this experiment? Is there a control group for both - within population analysis and between population analysis?
This was not really a hypothesis driven experiment, so there is no control group as such. This is more analogous to exploratory data analysis in fields like economics, where you collect some explanatory variables and some outcomes and look for statistical connections between them. Unlike economics, we do have the ability to do controlled experiments after the fact, and this would be an interesting next step. For example, we have proposed that diet changes development along the first principal component. A follow up hypothesis driven experiment could set N2 fed *E. coli* as the control group and assign a variety of bacterial diets as treatment groups. We could then assess statistically the effect of these new diets under our hypothesis. In this analysis, we have not assigned a single group as a control group but have

instead chosen to use tools such as linear regression models, which take into account all of the pairwise comparisons between groups.

10. Fig 2 (b) is the normalized data, but after normalization all conditions look the same (is this expected?)
    This is unexpected in the sense that it is not a trivial feature of the rescaling procedure used, that it doesn't have to be this way. If the data were different, it could be that the rescaled curves do not collapse. However, for our data, this was expected based on other studies of the timing of molecular events during development which can also be rescaled as cited in the main text (Filina *et al*, 2022).
11. What data is being read into the NLPCA? How many dimensions of data? Number of rows/samples? Number of columns/features? Are the data numerical? Continuous or discrete?
    673 samples for 5 features give a (673x5) input matrix, which is reduced by NLPCA to a (673x3) latent space projection. The data are all numerical and continuous.
12. Why specifically did they use NLPCA? Did they compare their results to other methods? Regular PCA? UMAP? Tsne?
    We chose NLPCA because we did not expect there to be linear relationships between the phenotypic parameters we measured. When we first looked at the 3d scatter plot of logistic fit parameters it looked like a curved plane. Some of the simplest physical laws in thermodynamics, for example the ideal gas law, have a nonlinear relationship between the variables of interest, i.e. $PV = nRT$ for P, V and T. With this in mind, and because NLPCA is more general, and able to find linear relationships as well if they exist, we chose this over PCA. It does seem that there is some non-linear relationship between the variables, as NLPCA performs better than regular PCA, as shown in the cumulative variance explained plot below.

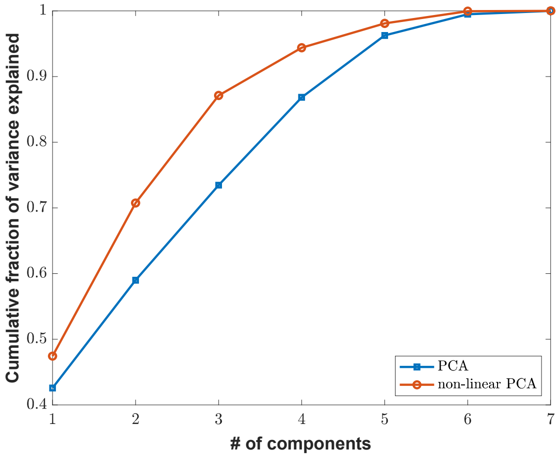

We did not choose t-SNE because it is not density preserving. This is clearly presented Wattenberg *et al.* (Wattenberg, et al., "How to Use t-SNE Effectively", Distill, 2016. http://doi.org/10.23915/distill.00002) which I quote below.

Because we were particularly interested in how the data clustered (See explanation of clustering and probability density functions in response to your question 20 below) after dimensionality reduction we decided not to use t-SNE.
The UMAP technique relies on an assumption we did not believe to be true for our data, i.e., that the data are uniformly distributed on Riemannian manifold, although it is difficult to assess how close to uniformly distributed the data truly are. In addition, while I can draw mathematical analogies between projection operators and both PCA and NLPCA, the relationship between projections and t-SNE or UMAP were not clear to me. I have included this discussion in the Appendix to the manuscript.

13. An advantage of nonlinear PCA over linear PCA is the ability to incorporate nominal and ordinal variables, and that it can handle and discover nonlinear relationships between these variables. Are the authors using any categorical data here?
We do not include any categorical data here. We chose nonlinear PCA mainly for discovering nonlinear relationships between the variables of interest.
14. Page 8 Results/Fig 4a - The authors state that NLPCA was used to determine the shape of this ambient space of rescaled logistic parameters with the developmental duration included. It is unclear to me what data was used in the NLPCA analysis - how can you feed in logistic parameters to perform any sort of PCA?
For the NLPCA analysis, a 5-vector consisting of the logistic fit parameters and developmental durations $(l_{max}, r, A, t_{dev}, t_{hatch})$. This was compressed into a 3-vector (ϕ_1, ϕ_2, ϕ_3) using the NLPCA procedure.
In order to demonstrate how you can feed logistic parameters in to perform a PCA, let me provide a concrete example using a 2d ambient space, a 1d manifold, and linear PCA. Let us start with a collection of growth curves generated by the logistic function with parameters (l_{max}, r, A). For simplicity, I will fix the A parameter in all of the growth curves.

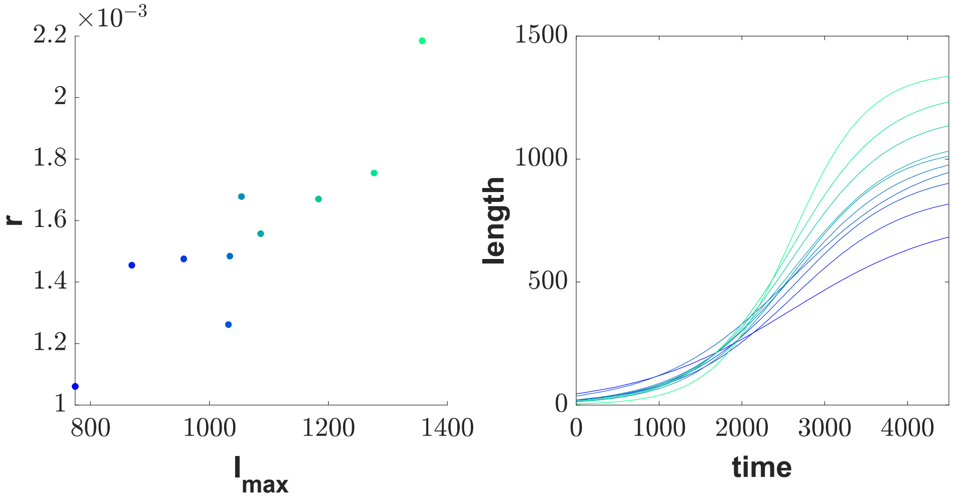

Because these two parameters are correlated, we can perform PCA and reduce the 2-vector (l_{max}, r) to a single scalar ϕ_1 by projecting it onto the first principal component.

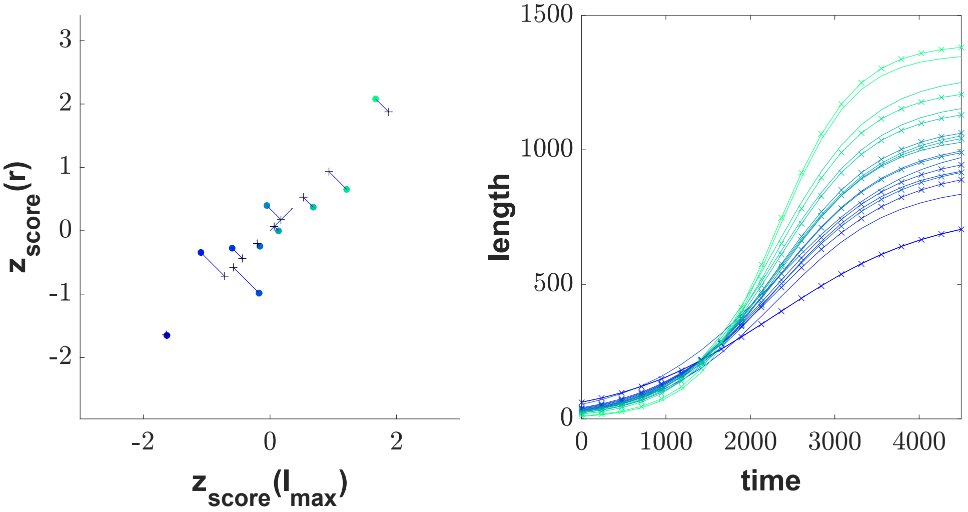

The main eigenvector (first principal component) is shown by the blue arrow and points in the [1,1] direction, $v = \left(\sqrt{(2)}, \sqrt{(2)}\right)$. The z-score of data points (centred scaled data) are show in the same colours as above. Their projections onto the first principal component are shown by the pluses.

l_{max}	r	ϕ_1	Reconstructed l_{max}	Reconstructed r
774.1	1.061e-03	-1.6404	772.1	1.064e-03
869.2	1.455e-03	-0.7174	935.4	1.342e-03
956.6	1.475e-03	-0.4364	985.2	1.427e-03
1031.9	1.262e-03	-0.5782	960.1	1.384e-03
1034.3	1.484e-03	-0.2021	1026.7	1.497e-03
1053.8	1.678e-03	0.1746	1093.3	1.611e-03
1086.2	1.557e-03	0.0657	1074.0	1.578e-03
1183.4	1.670e-03	0.5274	1155.8	1.717e-03
1276.9	1.755e-03	0.9322	1227.4	1.839e-03
1357.8	2.185e-03	1.8748	1394.2	2.123e-03

The results of reconstructing the growth curves from the compressed representation are shown by the "-x' lines in the right panel. I have included this discussion in the Appendix for the manuscript.

15. Could the authors also explain what an "ambient phenotypic space" is?
The "ambient phenotypic space" is the space of all phenotypic parameters that are measured and included in the analysis. I have included a paragraph in the introduction describing it in more detail. Briefly, it is vector of measurements before any dimensionality reduction is performed. Mathematically, it is a higher dimensional space into which a lower dimensional manifold is embedded. An example is the 2d surface of a sphere embedded into a 3d space.
16. Figure 4(a) - Could the authors elaborate more on figure 4a? This seems to be the major result of this paper, yet they dedicate only 1 - 2 sentences toward this result. What do the x,y,z, axis represent? Shouldn't they represent the principal component dimensions? Are these the first three extracted non-linear components?
Figure 4A shows the ambient space of 3 of the 5 parameters fed into the NLPCA. The x,y, and z axes are rescaled logistic fit parameters. It is not really a main result because it is only a cross section of the visualisable 5d space. The principal component dimensions are not the axis of this plot, but rather would be the axes depicted by the grid on the 2d sheet that cuts through the data in this figure.
17. FIgure 4(a) Could the authors elaborate on each principal component, by providing some sort of interpretation in the context of the data being used?
Figure 4 F and G provide an interpretation of the first two principal components. These panels show how variations in principal component scores result in different growth curves.
Figure 4(a) the figure caption says 2-D manifold, but the plot is 3-D.
The plot is 3d because the ambient space is 3d, and the manifold, which is the curved "sheet" plotted as mesh grid in this space, is 2d.
18. What are the curves in Fig 4b? What does the y - axis range represent?
    a. Are these the latent space projected onto 2D space?

    The curves in Fig4b are probability density functions given the probability that an individual takes that value of Phi_1 (score for the first nonlinear principal component). These are estimated using the kernel density method. I have included a new section in the methods describing in detail both probability density functions and the kernel density estimator. These can be found starting on line 523 in the revised MS.
19. Fig 4 (e) legend says "mixed mixed" regression - what is "mixed mixed" regression?
This was a typo, and I have changed the wording to make it clearer. Updated Figure Legend is on line 806.
20. Page 8 Fig 4 - "Within the embedding of growth curves, animals tend to cluster in phi_1 according to their food source" Is 4(b) meant to illustrate clusters? What is the meaning of a cluster in this context? Clusters usually means groups of individuals behaving in the same way.

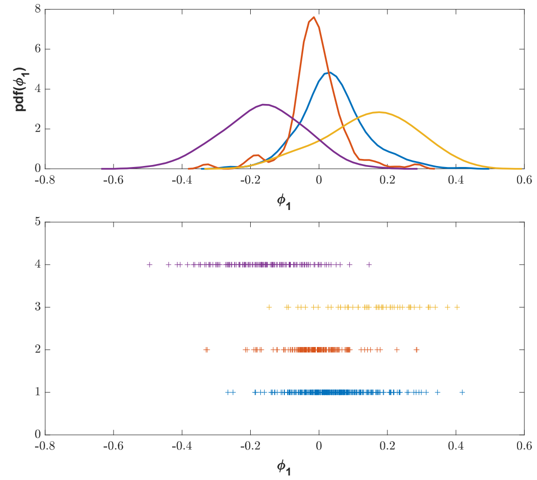

We do indeed mean “groups of individuals behaving in the same way” when we say clusters here. For clarification, I have plotted above the “behaviour” which is the location along the $\phi_1$axis for the four “groups of individuals” which are animals fer *E. coli* (blue) animals fed *Comamonas* (orange) animals fed *Pseudomonas* (purple) and animals fed *Bacilius* (yellow). They are separated on the y axis just for visual clarity. To behave in the same way, or to cluster, means that the individuals within a group are close that the individuals between groups. This I most obvious for the purple and yellow groups. They clearly “cluster” by eye. We can make this more quantitative by looking at their distributions, which are shown in the top panel of the above Figure. Clustering can be defined as having non-overlapping distributions, which we could define quantitatively in a number of ways, for example we could compute the relative entropy between the distributions. In this work we chose to compute the F-statistic, which compares the variance in the means, the mean of the variances. For the example of the purple and yellow distributions above, the difference in means is over 2.5 times the average of their standard deviations. This is quantitative evidence that these two conditions cluster separately from each other. The F-statistic displayed in Fig 4E takes into account all of the pairs of groups at once. Thus, it can be viewed as a sort of “degree of clustering” for each variable based on the groups.

21. Figure 4: What is the meaning of phi_1 and phi_2?

    Phi_1 and Phi_2 represent the first and second non-linear principal components and in the language of linear PCA, the plot in Figure 4D is a scatterplot of the *scores* of data points. These are the latent space projected in 2d. If this were linear PCA, these would be the weights of the first two eigenvectors.

22. Conclusion states that "Correlation between traits indicate that the true dimensionality of the system may be lower" but how is such a claim supported in this manuscript?

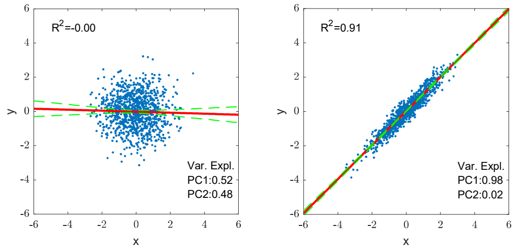

The statement that correlations between traits implies a lower dimensionality is not a statement based on conclusions drawn from our data *per se* but is a more general statement about the nature of the effective dimensionality of a set of variables and correlations between those variables. This is demonstrated in the figure above, where I have provided a scatter plot of two arbitrary variables x and y. In this example, x and y are not correlated in the left panel, which can be seen in the scatter plot and the correlation coefficient.

In contrast, the two variables x and y in the right panel are highly correlated with an r-squared of 0.91. If we consider the effective dimensionality of these two data sets, the left data cannot be reduced, and we see that the variance is almost equally distributed between the principal components. Thus, the uncorrelated 2d data remains 2d. However, on the right, there is significant correlation and the data are effectively 1d. The first principal component explains 98% of the variance. This argument also extends to higher dimensions. The use of r-squared as a correlation coefficient and PCA as a dimensionality reduction technique implies a linear relationship between the data, but other more general correlation coefficients can be used in conjunction with nonlinear dimensionality reduction techniques if nonlinear relationships are expected. For example, the correlation coefficient from:
Chatterjee S (2021) A New Coefficient of Correlation. J Am Stat Assoc 116: 2009–2022

23. The authors claim that genetic variation on the phenotype are separable from those of environmental variation. How is this true?

This is a statement about how well the data cluster based whether they are assigned to a group based on genotype (N2, AB1, or PS2025) or assign them based on their diet (E.coli, Comamonas, Pseudomonas, Bacillus). As I showed above in Answer 20, if we assign individuals to groups based on diet, we can cluster them using only their ϕ_1 value and we don't need ϕ_2. In contrast, we cannot cluster them well using any of the other single parameters. Alternatively, if we assign groups based on genotype, we can cluster them using only their ϕ_2 value. This is what we mean by separable.

Regarding the outcome of this study:

24. What was the actual outcome of this study? Did the authors identify the dominant mode of phenotypic variation?

Yes the dominant mode of phenotypic variation seems to be a tradeoff between faster growth and shorter adult length as shown in Fig 4F. This mode is captured quantitatively as the ϕ_1 value of each individual. This mode accounts for almost half of the variance between individuals and is primarily modulated by diet.

25. The authors don't compare to other methods, nor elaborate on how this is more widely applicable

We mention other experimental methodologies that attempt to understand robustness and plasticity including the work of (Sieriebriennikov & Sommer, 2018) (line 286), (Susoy *et al*, 2015) (line 330). It is difficult to compare methodologies directly because we are using different phenotypes, they study a morphological one and I a developmental one. However, other groups have studied development in the context of growth rates, and we compare out results to those obtained by (Stojanovski, 2022) and find that our results agree with their finding of a negative correlation between growth rate and developmental duration (Line 199) which they obtain using different methods. We also compare our results looking at correlations between traits to other groups who have found similar results in *Arabadopsis* (Pigliucci & Hayden, 2001) (Line 318) with results from the greenfinch that do not agree with our findings (Merilä & Björklund, 1999), and we speculate as to how differences in our methods may account for these contradictory findings (Line 320). Finally, we try to address the wider applicability by referencing connections to Statistical mechanics, (Lines 341, 347, 349) to gene expression dynamics (Lines 369-373), to sloppy parameter models (Line 365), to models of protein elasticity (Line 292 and 362) and to models of epigenetic inheritance (Lines 306 and 375).

Questions about methods/data/experimental conditions:

26. Can the authors show the data? The measured variables/prediction variables? Is this available?
Is the matlab code available for the NLPCA analysis?

The MATLAB code for all of the analysis is now provided as source data files and separately in a GitHUb repository at https://github.com/davex0r/DevelopmentalManifold. The NLPCA was performed using the nlpca package written by Mathias Scholz, which was described in the methods and referenced, in the current version this is the paragraph starting on Line 512 title Non-linear PCA and reference #46 (Scholz *et al.* 2005).

Is the raw data and normalized data available?

The raw and normalized data is available both as source data files in the current submission and on the GitHub repository

27. Can the authors provide a table which summarizes the variables/measurements being used in this study? Which variables are being measured manually or automatically, which variables are derived from logistic fit?
    We have provided a new table in the methods title Measurement Summary Table, on line 552 with all of the details.
28. In the results it states that length and area are measured automatically, but egg hatching and first laying egg are recorded manually? Which variables are recorded manually versus automatically?
    Length and area are measure automatically, egg-hatching time and first egg-laying time are recorded manually.
    a. How many variables are being measured and recorded? Two?
       We have provided a new table in the methods title Measurement Summary Table, on line 552 with all of the details.
    b. Is reproductive maturation another variable? If so, what is this a measure of?
       This is the time it takes for an embryo to mature ex-utero, hatch, grown through 4 larval stages, develop a functioning germline, fertilize an oocyte of its own, and lay the fertilized egg.
    c. Are the perturbations/conditions "natural genetic variation" and "single gene mutant"? Are these the two main environmental conditions?
       These are the two main genetic perturbations, a “condition” is a combination of genetic perturbation and diet perturbation.
    d. Is "wild isolates"/"natural selection another condition?
       Wild isolates for *C. elegans* are the same as natural genetic variation, the terms are used interchangeably.
    e. Diet conditions: different kinds of bacteria
       The diet conditions are indeed different kinds of bacteria being fed to the *C. elegans*

Minor points:

29. In the introduction (line numbers are not present) it says ..."and manually recording" - should this be "recorded"?

Line 141 Changed the following text: *Manually recording* to *manually recorded*

30. Fig 1 - are there 8 or 10 time points in (a) ? I only see 8 time points.

Line 740 Changed *10* to *8*

31. Fig 1(c) says 10 specific points highlighted, but I only see 8 points.

Line 749 Changed *10* to *8*

32. Page 5 says "differ food sources differ..." Should it say "different"?

Line 194 Changed the following text: *differ* to *different*

33. Box 2: do the authors cite the method (discrete cosine transformation) they use?

Line 871 Added text: *and implemented by the dct2 function in MATLAB*

34. Are the authors making a connection between Box 2 and canalisation/plasticity of c elegans?
35. Fig S2: (b) shouldn't the means (+) be different colors? Shouldn't the colors correspond to the colors in (a).

Yes, this has been fixed in the updated version of what is now Figure EV3

36. Fig S3: (a) how many individuals? (b) What are we supposed to be able to see in the heatmap/spatial distributions?

As this section was confusing and not necessary to understand the main text, I have removed it in this submission.

37. Is there a table explaining all the different conditions? (see legend showing conditions) in Fig S3 (a).

Line 555 I have added a table explaining all of the different conditions, title *Experimental Conditions Summary Table.*

38. What do the different colors represent in Figure S2 (a)?

These represent different diets. In the updated version of Figure EV4, there is a legend in panel B which gives the diet conditions and is shared between the panels. This has been noted in the caption.

39. The authors use phrases such as "cryptic variation" (see Discussion), what does this mean?
    We have added the following at line 280: *i.e. genetic variation that does not result in phenotypic differences under normal conditions, but can be revealed in certain circumstances,* to explain cryptic

variation.

"Incredibly diversity" in the Conclusion must be a typo?

Line 392 Changed text: *incredibly* to *incredible*

Reviewer 3

In the paper the authors study the homeorhesis of development of C elegans. By studying the growth curve on various conditions and mutants, they unveiled low-dimensional phenotypic manifold. The results are interesting, and the experimental analysis is clearly presented. I recommend the publication of the manuscript after the authors consider the following points. Some comments are a bit idiosyncratic, so these are for optional for revision of the present manuscript, but maybe for future studies.

1. The term homeorhesis was originally coined by Conrad Waddington, together with his renown landscape, so it will be good to mention this explicitly. Also, there are some theoretical studies for homeorhesis in developmental process (e.g., ref [1]).

   Lines 35-37

   Added the following text:

   terms introduced by CH Waddington in his series of foundational works on the development.

   Added the following references:

   (Waddington, 1942)

2. In contrast to the parameters l^{hat} and r^{hat} thoroughly investigated, the parameter A was less discussed. It will be good to discuss a bit more the meaning of A. At around t\sim A, the growth speed is maximal. Is there some biological meaning for it? Compared with l^{hat} or r^{hat}, how about is the degree of robustness (or variance) in A?

   We find that, in addition to maximal length, A is highly correlated with length at hatching. This might mean that the embryos already have some growth advantage at hatching. This is true for the sid-2 mutant, and may be due to some partitioning of extra yolk proteins from the intestine to the embryos before fertilisation. It would be interesting to explore this further, especially with higher-resolution imaging to capture variation in length at hatching better

3. Does the magnitude of variance of l just simply increase with development time? Or is there some bottleneck time as discussed in the hourglass picture? (This may depend on the way to select egg, though. --I wonder if the initial variance in natural condition may be larger and can be buffered in the middle stage in development).

   Interestingly, there does seem to be some sort of bottleneck or hourglass in the length variance, which occurs at the time of the onset of egg laying. In the figure below, I have plotted the relative variation of the length $\frac{l-\bar{l}}{\bar{l}}$ at four different developmental time points, hatching, the length at A (the time when growth is maximum), the length at the onset of egg-laying, and the asymptotic adult length (max length). The variance appears to decrease until the onset of egglaying, where it reaches its minimum and then increases again. I have not taken into account carefully the propagation of uncertainty for the variance in length at hatching, as the worms are smaller and the resolution of the imaging is relatively poorer at this stage, but even the variance at A is larger, where this should not be a problem. This requires further investigation, and I could not find a way to include it coherently in the current version of the manuscript, but it may form the basis for some future work. Thanks!

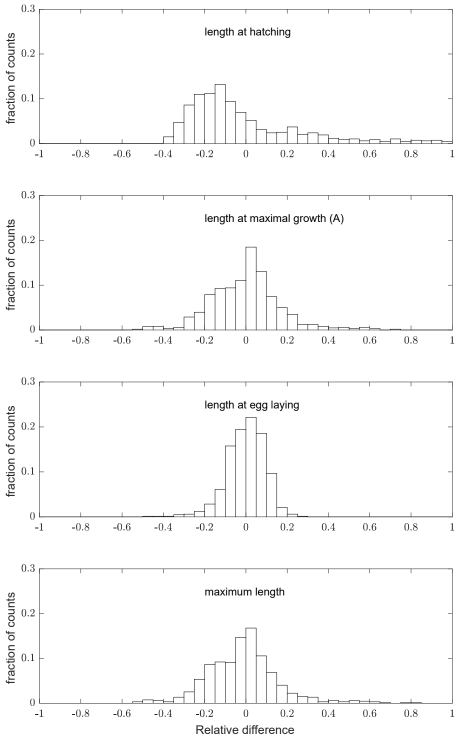

4. In the discussion, the authors mention that the origin of low-dimensional dynamics and of the fluctuation-response relationship remains a mystery. I agree that it is not solved. Still, there is some discussion on it, albeit it may be hand-waiving. In Furusawa and Kaneko ([2],) it is discussed, by assuming the robustness of the developed phenotype to perturbations, strong attraction to the state follows, while plasticity is kept in the direction of evolution, as the phenotype should change through the evolution. Then, the variances by noise (or environment) and by mutation follow the common manifold, leading to the correlation (proportionality)between the two [3], from which evolutionary fluctuation relationship follows.
   Now, in the present study, from the nonlinear PCA, the direction for plastic change is extracted, so that one may consider in which direction evolutionary plasticity is kept. Such discussion may be of biological significance.
   Indeed, there is some interesting discussion of this relationship! I did not mean to imply that it is totally mysterious, so I have changed the text to now read "While the origin of low dimensional dynamics and of fluctuation-dissipation like relations in biological systems has not been completely solved". And have included the reference to Furasawa and Kaneko [2] and [3] which I had missed! Thanks for bringing them to my attention. I have included these as references 17 and 18 in the main text.
5. Also, in the above sense, it will be interesting to explore possible relationship between the phenotypic variance by noise and by mutation (even though the phenotypes measured here are just three; l^{hat}, r^{hat} and A).
   In response to this and to the question of Reviewer 1 above, I have included a new figure which explores two mutations. I reproduce the response here for completeness.
6. I had originally neglected an in-depth analysis of the mutants, as I had not tested them on all the different bacterial diets, only the lab strain of *E. coli*. However, in preparing my reply to the reviewer's questions, I have found some very interesting results regarding the mutants and have included this analysis as a new Figure 5. The two mutants that I included were a deletion of the sid-2 gene, which is an otherwise healthy animal and whose main phenotype is the loss of the ability to do RNAi by feeding. We had previously seen that this mutation slightly accelerated development and led to slightly longer animals (but not as

long as long mutants such as *lon-2*). The other mutant is a deletion in a putative helicase that had a severe developmental delay with worms much smaller and "sicker". Interestingly, this severe mutant does indeed break trends in diet and genotype. Although the mild mutant overlaps significantly with the distributions for other N2 worms and for worms fed *E. coli*, the C28 mutant is very far from other N2 worms and from other worms fed *E. coli*. In addition, if we look at how "off manifold" the mutants are, by measuring their average position along the third nonlinear principal component ϕ_3 in fact we do find that both mutants are off the manifold (positive in the ϕ_3 direction). It is interesting to note that the *sid-2* mutant is off-manifold in the same way as the C28 mutant despite it having a mild phenotype. The wild-isolate strains AB1 and PS2025 are also slightly off-manifold, but in the opposite direction (negative ϕ_3) to the mutants! Furthermore, while the mutants are off-manifold when fed on E. coli, the wild-isolates are only off-manifold when their diets are perturbed as well. In addition to the new Figure 5 and the caption of the figure, I have included a paragraph in the results that describes these findings, as I have related them to you above.

7. I am not completely sure if the Box 2 is necessary. Still probably it will be useful to most biology readership, so I will leave the judgment to the editor.
   On advice of the other reviewer and the editor I have left this in.
8. The discussion on possible relationship in gene expression oscillator is interesting. According to the experiment the period is about 8 hours. It is smaller than A\sim 30hours, but in their experiment oscillation ceases by SNIC bifurcation after about a 4-5 periods or so. It may be useful explore this point, if possible. (In fact in ref [1], indeed, oscillatory dynamics provide homeorhesis).
   It will be indeed interesting to explore the connection between oscillatory dynamics and Homeorhesis further especially in the context of projection operators in Hilbert spaces. Thanks also for the reference, I have included it as reference 31 in the main text and on Line 375 in the revised manuscript.
9. References

   [1]Matsushita et al., Phys. Rev. Research 2, 023083(2020))
   [2]Furusawa and Kaneko, Physical Review E 97.4 (2018): 042410.
   [3]Furusawa and Kaneko, Journal of The Royal Society Interface 12.109 (2015): 20150482.

14th Sep 2023

Manuscript Number: MSB-2023-11835R
Title: Canalisation and plasticity on the developmental manifold of Caenorhabditis elegans

Dear Dr. Jordan,

Thank you for sending us your revised manuscript. We have now heard back from the two reviewers who agreed to evaluate your revised study. As you will see below, the reviewers are satisfied with the performed revisions and support publication. As such, I am glad to inform you that we can soon accept your manuscript for publication, pending some minor revisions listed below, all related to editorial issues.

- Our data editors have noticed some unclear or missing information in the figure legends, please see the attached .doc file. Please make all requested text changes using the attached file and *keeping the "track changes" mode* so that we can easily access the edits made.

- The References should be formatted according to the Molecular Systems Biology reference style (i.e. ordered alphabetically and listing the first 10 authors followed by et al).

- The Data Availability section should be moved after the Materials and Methods.

- Please include callouts for Fig 1D and for all Appendix figures (S1-S5).

- Please include a Table of Contents in the beginning of the Appendix.

- The synopsis image is rather large and detailed and not all labels display well at the final size required. Please resupply the image as a jpg or png at the required final size (it needs to be exactly 550 px wide, and the height ideally < 500 px), ensuring that all labels are legible.

Please resubmit your revised manuscript online, with a covering letter listing amendments and responses to each point raised by the referees. Please resubmit the paper **within one month** and ideally as soon as possible. If we do not receive the revised manuscript within this time period, the file might be closed and any subsequent resubmission would be treated as a new manuscript. Please use the Manuscript Number (above) in all correspondence.

Click on the link below to submit your revised paper.

https://msb.msubmit.net/cgi-bin/main.plex

As a matter of course, please make sure that you have correctly followed the instructions for authors as given on the submission website.

Thank you for submitting this paper to Molecular Systems Biology.

Kind regards,

Maria

Maria Polychronidou, PhD
Senior Editor
Molecular Systems Biology

If you do choose to resubmit, please click on the link below to submit the revision online before 14th Oct 2023.

https://msb.msubmit.net/cgi-bin/main.plex

IMPORTANT:
Please note that corresponding authors are required to supply an ORCID ID for their name upon submission of a revised manuscript (EMBO Press signed a joint statement to encourage ORCID adoption).
(https://www.embopress.org/page/journal/17444292/authorguide#editorialprocess)

Currently, our records indicate that the ORCID for your account is 0000-0002-3506-5440.

Please click the link below to modify this ORCID:
Link Not Available

The system will prompt you to fill in your funding and payment information. This will allow Wiley to send you a quote for the article processing charge (APC) in case of acceptance. This quote takes into account any reduction or fee waivers that you may be eligible for. Authors do not need to pay any fees before their manuscript is accepted and transferred to the publisher.

As a matter of course, please make sure that you have correctly followed the instructions for authors as given on the submission website.

*** PLEASE NOTE *** As part of the EMBO Press transparent editorial process initiative (see our Editorial at https://dx.doi.org/10.1038/msb.2010.72 , Molecular Systems Biology will publish online a Review Process File to accompany accepted manuscripts. When preparing your letter of response, please be aware that in the event of acceptance, your cover letter/point-by-point document will be included as part of this File, which will be available to the scientific community. More information about this initiative is available in our Instructions to Authors. If you have any questions about this initiative, please contact the editorial office (msb@embo.org).

Reviewer #1:

I think the new analysis adds to the paper. Given the other reviewers' comments and the authors' response I remain enthusiastic about the paper.

Reviewer #3:

I recommend the publication of the manuscriptMSB-2023-11835R by David Jordan and E Miska.
The revised manuscript has fully addressed my earlier comment, and the manuscript is much improved to be publishable in MSB.

All editorial and formatting issues were resolved by the authors.

5th Oct 2023

Manuscript number: MSB-2023-11835RR
Title: Canalisation and plasticity on the developmental manifold of Caenorhabditis elegans

Dear Dr. Jordan,

Thank you again for sending us your revised manuscript. We are now satisfied with the modifications made and I am pleased to inform you that your paper has been accepted for publication.

*** PLEASE NOTE *** As part of the EMBO Publications transparent editorial process initiative (see our Editorial at https://dx.doi.org/10.1038/msb.2010.72), Molecular Systems Biology publishes online a Review Process File with each accepted manuscripts. This file will be published in conjunction with your paper and will include the anonymous referee reports, your point- by-point response and all pertinent correspondence relating to the manuscript. If you do NOT want this File to be published, please inform the editorial office at msb@embo.org within 14 days upon receipt of the present letter.

Should you be planning a Press Release on your article, please get in contact with msb@wiley.com as early as possible, in order to coordinate publication and release dates.

LICENSE AND PAYMENT:
All articles published in Molecular Systems Biology are fully open access: immediately and freely available to read, download and share.

Molecular Systems Biology charges an article processing charge (APC) to cover the publication costs. You, as the corresponding author for this manuscript, should have already received a quote with the article processing fee separately. Please let us know in case this quote has not been received.

Once your article is at Wiley for editorial production you will receive an email from Wiley's Author Services system, which will ask you to log in and will present you with the publication license form for completion. Within the same system the publication fee can be paid by credit card, an invoice or pro forma can be requested.

Payment of the publication charge and the signed Open Access Agreement form must be received before the article can be published online.

Molecular Systems Biology articles are published under the Creative Commons licence CC BY, which facilitates the sharing of scientific information by reducing legal barriers, while mandating attribution of the source in accordance to standard scholarly practice.

Proofs will be forwarded to you within the next 2-3 weeks.

Thank you very much for submitting your work to Molecular Systems Biology.

Kind regards,

Maria

Maria Polychronidou, PhD
Senior Editor
Molecular Systems Biology

EMBO Press Author Checklist

Corresponding Author Name: David J Jordan	
Journal Submitted to: Molecular Systems Biology	
Manuscript Number: MSB-2023-11835	

USEFUL LINKS FOR COMPLETING THIS FORM
The EMBO Journal - Author Guidelines
EMBO Reports - Author Guidelines
Molecular Systems Biology - Author Guidelines
EMBO Molecular Medicine - Author Guidelines

Reporting Checklist for Life Science Articles (updated January
This checklist is adapted from Materials Design Analysis Reporting (MDAR) Checklist for Authors. MDAR establishes a minimum set of requirements in transparent reporting in the life sciences (see Statement of Task: 10.31222/osf.io/9sm4x). Please follow the journal's guidelines in preparing your
Please note that a copy of this checklist will be published alongside your article.

Abridged guidelines for figures
1. Data
The data shown in figures should satisfy the following conditions:
- the data were obtained and processed according to the field's best practice and are presented to reflect the results of the experiments in an accurate and unbiased manner.
- ideally, figure panels should include only measurements that are directly comparable to each other and obtained with the same assay.
- plots include clearly labeled error bars for independent experiments and sample sizes. Unless justified, error bars should not be shown for technical
- if n<5, the individual data points from each experiment should be plotted. Any statistical test employed should be justified.
- Source Data should be included to report the data underlying figures according to the guidelines set out in the authorship guidelines on Data

2. Captions
Each figure caption should contain the following information, for each panel where they are relevant:
- a specification of the experimental system investigated (eg cell line, species name).
- the assay(s) and method(s) used to carry out the reported observations and measurements.
- an explicit mention of the biological and chemical entity(ies) that are being measured.
- an explicit mention of the biological and chemical entity(ies) that are altered/varied/perturbed in a controlled manner.
- the exact sample size (n) for each experimental group/condition, given as a number, not a range;
- a description of the sample collection allowing the reader to understand whether the samples represent technical or biological replicates (including how many animals, litters, cultures, etc.).
- a statement of how many times the experiment shown was independently replicated in the laboratory.
- definitions of statistical methods and measures:
  - common tests, such as t-test (please specify whether paired vs. unpaired), simple χ2 tests, Wilcoxon and Mann-Whitney tests, can be unambiguously identified by name only, but more complex techniques should be described in the methods section;
  - are tests one-sided or two-sided?
  - are there adjustments for multiple comparisons?
  - exact statistical test results, e.g., P values = x but not P values < x;
  - definition of 'center values' as median or average;
  - definition of error bars as s.d. or s.e.m.

Please complete ALL of the questions below.
Select "Not Applicable" only when the requested information is not relevant for your study.

Materials

Newly Created Materials	Information included in the manuscript?	In which section is the information available? (Reagents and Tools Table, Materials and Methods, Figures, Data Availability Section)
New materials and reagents need to be available; do any restrictions apply?	Yes	Reagents and Tools Table

Antibodies	Information included in the manuscript?	In which section is the information available? (Reagents and Tools Table, Materials and Methods, Figures, Data Availability Section)
For **antibodies** provide the following information:<br>- Commercial antibodies: RRID (if possible) or supplier name, catalogue number and or/clone number<br>- Non-commercial: RRID or citation	Not Applicable	

DNA and RNA sequences	Information included in the manuscript?	In which section is the information available? (Reagents and Tools Table, Materials and Methods, Figures, Data Availability Section)
**Short novel DNA or RNA including primers, probes**: provide the sequences.	Not Applicable	

Cell materials	Information included in the manuscript?	In which section is the information available? (Reagents and Tools Table, Materials and Methods, Figures, Data Availability Section)
**Cell lines:** Provide species information, strain. Provide accession number in repository **OR** supplier name, catalog number, clone number, and/**OR** RRID.	Not Applicable	
**Primary cultures:** Provide species, strain, sex of origin, genetic modification status.	Not Applicable	
Report if the cell lines were recently **authenticated** (e.g., by STR profiling) and tested for mycoplasma contamination.	Not Applicable	

Experimental animals	Information included in the manuscript?	In which section is the information available? (Reagents and Tools Table, Materials and Methods, Figures, Data Availability Section)
**Laboratory animals or Model organisms:** Provide species, strain, sex, age, genetic modification status. Provide accession number in repository **OR** supplier name, catalog number, clone number, **OR** RRID.	Yes	Reagents and Tools Table
**Animal observed in or captured from the field:** Provide species, sex, and age where possible.	Not Applicable	
Please detail **housing and husbandry conditions**.	Yes	Methods and Protocols

Plants and microbes	Information included in the manuscript?	In which section is the information available? (Reagents and Tools Table, Materials and Methods, Figures, Data Availability Section)
**Plants:** provide species and strain, ecotype and cultivar where relevant, unique accession number if available, and source (including location for collected wild specimens).	Not Applicable	
**Microbes:** provide species and strain, unique accession number if available, and source.	Yes	Reagents and Tools Table

Human research participants	Information included in the manuscript?	In which section is the information available? (Reagents and Tools Table, Materials and Methods, Figures, Data Availability Section)
If collected and within the bounds of privacy constraints report on age, sex and gender or ethnicity for all study participants.	Not Applicable	

Core facilities	Information included in the manuscript?	In which section is the information available? (Reagents and Tools Table, Materials and Methods, Figures, Data Availability Section)
If your work benefited from core facilities, was their service mentioned in the acknowledgments section?	Yes	Acknowledgements

Design

Study protocol	Information included in the manuscript?	In which section is the information available? (Reagents and Tools Table, Materials and Methods, Figures, Data Availability Section)
If study protocol has been **pre-registered, provide DOI in the manuscript**. For clinical trials, provide the trial registration number **OR** cite DOI.	Not Applicable	
Report the **clinical trial registration number** (at ClinicalTrials.gov or equivalent), where applicable.	Not Applicable	

Laboratory protocol	Information included in the manuscript?	In which section is the information available? (Reagents and Tools Table, Materials and Methods, Figures, Data Availability Section)
Provide DOI OR other citation details if **external detailed step-by-step protocols** are available.	Not Applicable	

Experimental study design and statistics	Information included in the manuscript?	In which section is the information available? (Reagents and Tools Table, Materials and Methods, Figures, Data Availability Section)
Include a statement about **sample size** estimate even if no statistical methods were used.	Yes	Experimental Conditions Sumamry Tabel (Methods and Protocols)
Were any steps taken to minimize the effects of subjective bias when allocating animals/samples to treatment (e.g. **randomization procedure**)? If yes, have they been described?	Not Applicable	
Include a statement about **blinding** even if no blinding was done.	Not Applicable	
Describe **inclusion/exclusion criteria** if samples or animals were excluded from the analysis. Were the criteria pre-established?<br><br>If sample or data points were omitted from analysis, report if this was due to attrition or intentional exclusion and provide justification.	Not Applicable	
For every figure, are **statistical tests** justified as appropriate? Do the data meet the assumptions of the tests (e.g., normal distribution)? Describe any methods used to assess it. Is there an estimate of variation within each group of data? Is the variance similar between the groups that are being statistically compared?	Yes	Figure Captions, Methods and Protocols

Sample definition and in-laboratory replication	Information included in the manuscript?	In which section is the information available? (Reagents and Tools Table, Materials and Methods, Figures, Data Availability Section)
In the figure legends: state number of times the experiment was **replicated** in laboratory.	Yes	
In the figure legends: define whether data describe **technical or biological replicates**.	Yes	

Ethics

Ethics	Information included in the manuscript?	In which section is the information available? (Reagents and Tools Table, Materials and Methods, Figures, Data Availability Section)
Studies involving **human participants**: State details of **authority granting ethics approval** (IRB or equivalent committee(s), provide reference number for approval.	Not Applicable	
Studies involving **human participants**: Include a statement confirming that **informed consent** was obtained from all subjects and that the experiments conformed to the principles set out in the WMA Declaration of Helsinki and the Department of Health and Human Services Belmont Report.	Not Applicable	
Studies involving **human participants:** For publication of **patient photos**, include a statement confirming that consent to publish was obtained.	Not Applicable	
Studies involving experimental **animals**: State details of **authority granting ethics approval** (IRB or equivalent committee(s), provide reference number for approval. Include a statement of compliance with ethical regulations.	Not Applicable	
Studies involving **specimen and field samples:** State if relevant **permits** obtained, provide details of authority approving study; if none were required, explain why.	Not Applicable	

Dual Use Research of Concern (DURC)	Information included in the manuscript?	In which section is the information available? (Reagents and Tools Table, Materials and Methods, Figures, Data Availability Section)
Could your study fall under dual use research restrictions? Please check biosecurity documents and list of **select agents and toxins** (CDC): https://www.selectagents.gov/sat/list.htm	Not Applicable	
If you used a select agent, is the security level of the lab appropriate and reported in the manuscript?	Not Applicable	
If a study is subject to dual use research of concern regulations, is the name of the **authority granting approval and reference number** for the regulatory approval provided in the manuscript?	Not Applicable	

Reporting

The MDAR framework recommends adoption of discipline-specific guidelines, established and endorsed through community initiatives. Journals have their own policy about requiring specific guidelines and recommendations to complement MDAR.

Adherence to community standards	Information included in the manuscript?	In which section is the information available? (Reagents and Tools Table, Materials and Methods, Figures, Data Availability Section)
State if relevant guidelines or checklists (e.g., **ICMJE, MIBBI, ARRIVE, PRISMA**) have been followed or provided.	Not Applicable	
For **tumor marker prognostic studies,** we recommend that you follow the **REMARK** reporting guidelines (see link list at top right). See author guidelines, under 'Reporting Guidelines'. Please confirm you have followed these guidelines.	Not Applicable	
For **phase II and III randomized controlled trials**, please refer to the **CONSORT** flow diagram (see link list at top right) and submit the CONSORT checklist (see link list at top right) with your submission. See author guidelines, under 'Reporting Guidelines'. Please confirm you have submitted this list.	Not Applicable	

Data Availability

Data availability	Information included in the manuscript?	In which section is the information available? (Reagents and Tools Table, Materials and Methods, Figures, Data Availability Section)
Have **primary datasets** been deposited according to the journal's guidelines (see 'Data Deposition' section) and the respective accession numbers provided in the Data Availability Section?	Yes	Source Data files and GitHub repository
Were **human clinical and genomic datasets** deposited in a public access-controlled repository in accordance to ethical obligations to the patients and to the applicable consent agreement?	Not Applicable	
Are **computational models** that are central and integral to a study available without restrictions in a machine-readable form? Were the relevant accession numbers or links provided?	Yes	GitHub repository
If publicly available data were reused, provide the respective **data citations in the reference list**.	Not Applicable	
